# Prevalence of depression, anxiety and suicide among men who have sex with men in China: a systematic review and meta-analysis

**DOI:** 10.1017/S2045796020000487

**Published:** 2020-06-15

**Authors:** D. Wei, X. Wang, X. You, X. Luo, C. Hao, J. Gu, S. Peng, X. Yang, Y. Hao, Vincent M. B. Silenzio, J. Li, F. Hou

**Affiliations:** 1 School of Public Health, Sun Yat-Sen University, No. 74, Zhongshan Second Road, Guangzhou, China; 2 School of Public Health, Lanzhou University, Lanzhou, China; 3 Evidence-based Medicine Center, School of Basic Medical Sciences, Lanzhou University, Lanzhou, China; 4 Sun Yat-Sen Global Health Institute, Sun Yat-Sen University, Guangzhou, China; 5 Department of Urban-Global Public Health, Rutgers School of Public Health, The State University of New Jersey, NJ, USA; 6 Department of Public Health, Shenzhen Kangning Hospital/Shenzhen Mental Health Center, Shenzhen, China

**Keywords:** Anxiety symptoms, China, depressive symptoms, men who have sex with men, suicide

## Abstract

**Aims:**

Chinese men who have sex with men (MSM) are at high risk for depression, anxiety and suicide. The estimated prevalence of these problems is essential to guide public health policy, but published results vary. This meta-analysis aimed to estimate the prevalence of depressive symptoms, anxiety symptoms and suicide among Chinese MSM.

**Methods:**

Systematic searches of EMBASE, MEDLINE, PsycINFO, PubMed, CNKI and Wanfang databases with languages restricted to Chinese and English for studies published before 10 September 2019 on the prevalence of depressive symptoms, anxiety symptoms, suicidal ideation, suicide plans and suicide attempts among Chinese MSM. Studies that were published in the peer-reviewed journals and used validated instruments to assess depression and anxiety were included. The characteristics of studies and the prevalence of depression and anxiety symptoms, suicidal ideation, suicide plans and suicide attempts were independently extracted by authors. Random-effects modelling was used to estimate the pooled rates. Subgroup analysis and univariate meta-regression were conducted to explore potential sources of heterogeneity. This study followed the PRISMA and MOOSE.

**Results:**

Sixty-seven studies were included. Fifty-two studies reported the prevalence of depressive symptoms, with a combined sample of 37 376 people, of whom 12 887 [43.2%; 95% confidence interval (CI), 38.9–47.5] reported depressive symptoms. Twenty-seven studies reported the prevalence of anxiety symptoms, with a combined sample of 10 531 people, of whom 3187 (32.2%; 95% CI, 28.3–36.6) reported anxiety symptoms. Twenty-three studies reported the prevalence of suicidal ideation, with a combined sample of 15 034 people, of whom 3416 (21.2%; 95% CI, 18.3–24.5) had suicidal ideation. Nine studies reported the prevalence of suicide plans, with a combined sample of 5271 people, of whom 401 (6.2%; 95% CI, 3.9–8.6) had suicide plans. Finally, 19 studies reported the prevalence of suicide attempts, with a combined sample of 27 936 people, of whom 1829 (7.3%; 95% CI, 5.6–9.0) had attempted suicide.

**Conclusions:**

The mental health of Chinese MSM is poor compared with the general population. Efforts are warranted to develop interventions to prevent and alleviate mental health problems among this vulnerable population.

## Introduction

Gay, bisexual and other men who have sex with men (MSM) appear to be disproportionately affected by a variety of psychological problems, including depression, distress, generalised anxiety disorder, substance use and suicide (Batchelder *et al*., 2017; King *et al*., 2008). According to Meyer's sexual minority stress model, the stressors induced by a homophobic environment specific to the sexual minority status can lead to psychological problems in sexual minorities (Meyer, 2003). Traditional Chinese culture promotes heterosexual marriage, procreation and filial piety, leading to low social acceptance of same-sex behaviours (Steward *et al*., 2013). Same-sex marriage, civil unions and other same-sex partnerships are not supported by law in China, nor are there relevant anti-discrimination laws and policies (Zhang and Chu, 2005; Cao and Guo, 2016). These social and cultural characteristics create a heteronormative and stigmatising environment for Chinese MSM, detrimental to their mental health. A recent review indicated that Chinese MSM have a high prevalence of several mental health issues, including depression, anxiety, suicidal behaviour and alcohol abuse, which can be explained by minority stress (Sun *et al*., 2020). These psychological problems are conducive to the ‘syndemic’ conditions surrounding human immunodeficiency virus (HIV) transmission risk behaviours and indirectly contribute to the high prevalence of HIV among MSM (Tsai and Burns, 2015; Safren *et al*., 2010).

Despite an influx of studies focusing on the prevalence of depression, anxiety and suicide among MSM in China, the reported prevalence varies due to methodologies and sample characteristics, indicating the importance of reliable estimates from a pooled prevalence of these psychological problems. With a decade of relevant studies available, enough data exist to obtain a summary prevalence and explore potential sources of heterogeneity via a meta-analysis. Reliable estimates of depression, anxiety and suicidal behaviour are important to identify the precise number of people with psychological problems, better understand the mental health status of the sexual minority population, and develop strategies to improve their psychological well-being. In addition, our work encourages the development of comprehensive strategies, based on psychosomatic medicine and social policies, against the HIV/AIDS epidemic among MSM.

Thus, we conducted a systematic review and meta-analysis of published studies on depressive symptoms, anxiety symptoms and suicide (including suicidal ideation, suicide plans and suicide attempts) among Chinese MSM. The purposes of this meta-analysis were to (1) estimate the prevalence of these psychological problems and (2) identify the underlying methodological and sample characteristic moderators that can contribute to between-study heterogeneity.

## Methods

We conducted this meta-analysis in accordance with the standards of Preferred Reporting Items for Systematic Reviews and Meta-analyses (PRISMA) and Meta-analysis of Observational Studies in Epidemiology (MOOSE) (Stroup *et al*., 2000; Moher *et al*., 2009).

### Search strategy

Two authors (DW and XY) performed a systematic search of the EMBASE, MEDLINE, PsycINFO, PubMed, CNKI and Wanfang databases, with languages restricted to Chinese and English for studies published before 10 September 2019, on the prevalence of depressive symptoms, anxiety symptoms, suicidal ideation, suicide plans and suicide attempts among Chinese MSM. In addition, the authors screened the reference lists of previous review articles on Google Scholar and Baidu Research to identify relevant studies that might have been missed, and then contacted the corresponding authors of these articles for missing information.

The database search combined terms related to MSM and study design with those related to depression or anxiety or suicidal ideation or suicide plans or suicide attempts, with the regional restriction of China. The keywords were selected based on each database (full details of the search strategy are provided in online Supplementary Table S1).

### Study eligibility

We included studies in the meta-analysis that (1) reported original quantitative data on one of our outcomes of interest related to gay, bisexual and other MSM (defined on the basis of self-identity, sexual behaviour or sexual attraction) in China; (2) were published in peer-reviewed journals and (3) used a standardised scale to assess depression and anxiety symptoms.

We excluded studies that (1) did not clearly define the study area in the original article; (2) could not extract the prevalence data by indirect calculation or by contacting the corresponding author; (3) that were conference papers, dissertations, reviews, case studies and qualitative studies and (4) the sample size was less than 30 people. The titles and abstracts of all identified studies were independently screened for eligibility by two authors (DW and XY), and any conflict was resolved by the first author of this study. The eligibility of all studies that successfully passed the title/abstract screening stage was similarly confirmed by the same two authors at the full-text screening stage.

### Data extraction

Using an extraction form piloted in three eligible studies, four authors (DW, XY, XY and SP) independently extracted the following data from each article: study design, survey year, study location, screening method used, population characteristics, sample size, recruitment, psychological outcomes and reported prevalence estimates. For longitudinal studies and intervention studies, only the baseline data were extracted. When more than one study used the same MSM sample, only the most comprehensive or most recent publication was included. Initial data extraction was checked by the first author. Immediately before the final analysis, all included data were checked again.

### Quality assessment

The Joanna Briggs Institute Critical Appraisal tool for use in JBI Systematic Reviews for prevalence studies (JBI checklist) was used to assess the trustworthiness, relevance and results of all included studies by two authors (DW and XY) independently (Munn *et al*., 2015). We made minor changes to the tool based on the study characteristics (see online Supplementary Table S2 for more details). The final checklist consisted of ten items with a score of 0 ‘not mentioned’, 1 ‘mentioned but not described in detail’ or 2 ‘detailed and comprehensive description’. The higher the total score, the better the quality of the study. Quality scores for all included studies are shown in online Supplementary Table S3.

### Data synthesis and analysis

The prevalence estimates with a 95% confidence interval (CI) of depressive symptoms, anxiety symptoms, suicide ideation, suicide plans and suicide attempts were calculated by pooling study-specific estimates using a random-effects meta-analysis accounting for heterogeneity (Borenstein *et al*., 2010). *Q*-tests and *I*^2^ were used to assess the level of heterogeneity between studies (i.e. the percentage of variability in prevalence estimates due to heterogeneity rather than sampling error or chance, with values ⩾75% indicating considerable heterogeneity) (Higgins and Thompson, 2002; Higgins *et al*., 2003). Univariate meta-regression and subgroup analysis based on study-level characteristics (study region, survey year, sampling method, study population, sample size, screening method and recall time for suicidality) were conducted to explore potential sources of heterogeneity. The regional classification was based on China's economic geographic division, including the first developing eastern region, the revitalised northeast region, the rising central region and the developing western region. Sensitivity analysis was performed sequentially, excluding each study, to determine the influence of individual studies on the overall prevalence estimates. Egger's test (Egger *et al.,* 1997*)* was used to test publication bias, *p* < 0.05 indicating statistically significant publication bias.^16^ All analyses were performed using R version 3.6.1 (with meta and metafor packages) (Schwarzer, 2007; Viechtbauer, 2010).

## Results

### Study characteristics

**Fig. 1.**
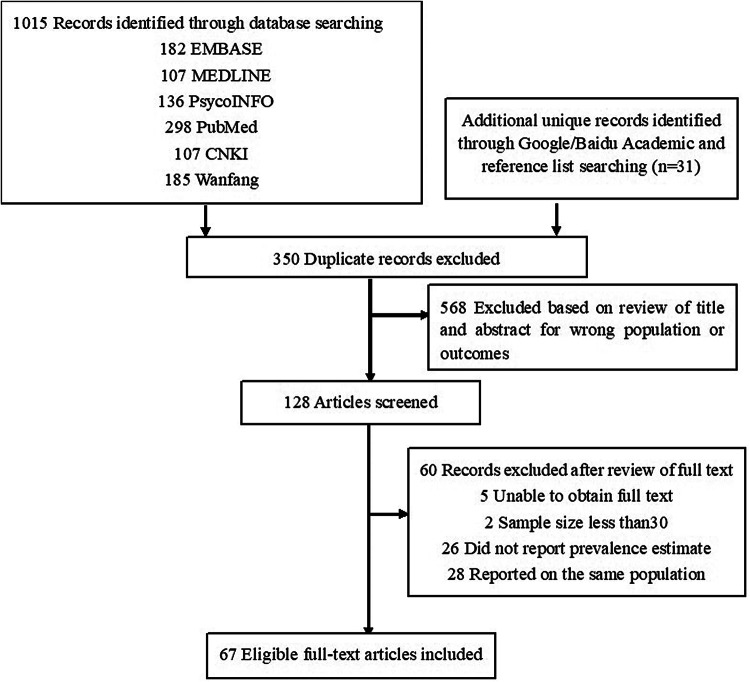
PRISMA flow diagram detailing the search strategy.

The PRISMA flow diagram outlines the search strategy used to identify the studies (Fig. 1). Sixty-seven studies were included in the final meta-analysis. Among them, 52 studies reported the prevalence of depressive symptoms, with a combined sample of 37 376 people. Twenty-seven studies reported the prevalence of anxiety symptoms, with a combined sample of 10 531 people. Finally, 23, 9 and 19 studies reported the prevalence of suicidal ideation, suicide plans and suicide attempts, with combined samples of 15 034 people, 5271 people and 27 936 people, respectively.

Among these studies, 37 were written in Chinese and 30 in English. In addition, 64 studies used a cross-sectional design, two used a cohort design (Tao *et al*., 2017*b*; Wang *et al*., 2018) and one used a randomised-controlled trial design (Tao *et al*., 2017*a*). The quality of the included studies varied considerably, with JBI scores ranging from 6 to 19 (mean score = 13).

The time span of these studies extended from 2001 to 2019, and the study population covered 15 provinces or cities (Shanghai, Beijing, Zhejiang, Guangdong, Jiangsu, Shandong, Tianjin, Liaoning, Anhui, Jiangxi, Hubei, Sichuan, Chongqing, Guangxi and Yunnan) and Taiwan. The sample size ranged from 50 to 15 066, and the target population included general MSM, young sexual minority males (YSMM), HIV-positive, HIV-negative, or serostatus-unknown MSM and money boys (MB, referring to male sex workers in China who engage in sex with men for economic survival). Forty-eight studies used behavioural measures to define their study population as MSM. Four studies (Sun *et al*., 2014*a*, 2014*b*; Yu *et al*., 2016; Xiong *et al*., 2018) used self-reported sexual orientation and five studies (Chen *et al*., 2012; Hu *et al*., 2016; Wang *et al*., 2016; Yu and Xiao, 2017; Gao *et al*., 2018) used both self-reported homosexual behaviour and sexual orientation to define their study population. In addition, four studies (Huang *et al*., 2010; Li *et al*., 2017; Huang *et al*., 2018*a*, 2018*b*) focused on sexual minority adolescents with two study populations, defining the study population using self-reported sex attraction. Six studies (Lv *et al*., 2014; Shiu *et al*., 2014; Yan *et al*., 2014; Chen *et al*., 2015; Tao *et al*., 2017*a*, 2017*b*) did not report how they identified their study population. Moreover, ten assessment methods were used to measure the prevalence of depressive symptoms, and seven were used to measure the prevalence of anxiety symptoms. The most common screening tools for depression were the Center for Epidemiologic Studies Depression Scale (CES-D) (Radloff, 1977) and the Zung Self-rating Depression Scale (SDS) (Biggs *et al*., 1978), used in 27 and 14 studies, respectively. Anxiety was mainly measured with the Self-rating Anxiety Scale (SAS) (Zung, 1971) and the Generalised Anxiety Disorder Scale-7 (GAD-7) (Spitzer *et al*., 2006), used in 17 and 4 studies, respectively. The measurement of suicide (suicide ideation, suicide plans or suicide attempts) based on a single item, except for two studies (Yu and Xiao, 2017; Yu *et al*., 2018), used standardised scales [a section of the World Mental Health Composite International Diagnostic Interview (WMH-CIDI)] (Kessler and Ustün, 2004) and a shortened 6-item version of the suicide questionnaire designed by Kessler *et al*. (2005). The recall time to measure suicide included ‘over the past week’, ‘over the past six months’, ‘over the past year’, ‘since HIV diagnosis’ and ‘lifetime’. The recruitment methods used in these studies included snowball sampling, respondent-driven sampling (RDS), recruitment through voluntary counselling and testing (VCT) clinics, online surveys and recruitment through gay-friendly non-governmental organisations. A summary of the characteristics of included studies is provided in online Supplementary Table S4.

### Depressive symptoms

Meta-analytic pooling of the prevalence of depressive symptoms reported in the 52 studies identified yielded a summary prevalence of 43.2% (12 887/37 376 individuals; 95% CI, 38.9–47.5), with significant evidence of between-study heterogeneity (*I*^2^ = 96.6%; Table 1). Sensitivity analysis showed that no individual study significantly affected the overall prevalence. In the subgroup analysis, heterogeneity was found to be reduced for the 2004–2007 period (*I*^2^ = 56.7%) in the MB population (*I*^2^ = 0.0%) and the YSMM population (*I*^2^ = 0.0%) for a sample size <100 (*I*^2^ = 70.6%), and in studies using the CESD-10 with a cut-off score of 10 or above (*I*^2^ = 29.9%) and HADS with a cut-off score of 8 or above (*I*^2^ = 29.7%) to identify significant depression.

**Table 1.** Estimated depression prevalence among MSM in China

				Subgroup analysis				Meta regression		
Subgroup	No. of studies	No. of depression	Sample size	Estimated rate	95% CI	*Q*	*I*^2^ (%)	*I*^2^ (%)	*Q*_m_	*p*
Study region								97.7	4.028	0.546
Central	4	348	737	0.502	0.412, 0.591	19.41	81.2			
Eastern	29	4620	10 629	0.447	0.383, 0.513	639.51	97.6			
Multiple	6	5665	20 514	0.378	0.302, 0.461	395.82	98.6			
Northeast	4	784	1859	0.403	0.237, 0.595	183.76	98.2			
Taiwan	1	154	620	0.248	0.216, 0.284	0.00	–			
Western	8	1316	3017	0.424	0.360, 0.491	98.27	92.1			
Survey year								97.8	5.848	0.211
2004–2007	4	595	1035	0.590	0.537, 0.640	9.40	56.7			
2008–2011	6	1510	3755	0.437	0.304, 0.581	294.3	98.5			
2012–2015	29	9089	28 594	0.407	0.367, 0.447	1216.4	96.8			
2016–2019	7	826	2094	0.431	0.241, 0.644	233.83	98.7			
*N*	6	867	1898	0.450	0.368, 0.534	89.26	91.3			
Sampling methods								97.1	11.755	0.109
Cluster	2	802	2483	0.280	0.228, 0.339	71.81	97.2			
Multiple methods	8	2431	6054	0.424	0.359, 0.493	119.59	96.1			
NGO	7	834	2446	0.363	0.273, 0.464	157.75	95.6			
RDS	8	1361	3159	0.403	0.319, 0.492	143.49	95.7			
Snowball	9	1230	2667	0.431	0.368, 0.496	109.54	91.4			
VCT clinic	15	2093	4571	0.472	0.366, 0.579	373.51	97.9			
Venue-based	2	175	283	0.623	0.546, 0.694	3.25	37.9			
Web	1	186	291	0.639	0.582, 0.692	0.00	–			
Study population								96.8	17.685	**0.007**
HIVMSM	16	2364	4877	0.505	0.407, 0.602	408.43	97.7			
HN/UnMSM	2	818	2316	0.323	0.252, 0.404	20.26	90.2			
HNMSM	2	141	447	0.292	0.205, 0.398	8.19	75.7			
MB	2	224	355	0.631	0.580, 0.680	1.85	0.0			
MSM	22	3910	9417	0.413	0.366, 0.462	396.73	95.4			
MSM, MB	2	742	1756	0.474	0.337, 0.616	51.41	96.1			
YSMM	6	4688	18 208	0.315	0.272, 0.361	101.4	90.8			
Sample size								97.8	5.051	0.282
<100	4	109	243	0.439	0.326, 0.560	13.10	70.6			
100–300	15	1479	2859	0.497	0.386, 0.608	291.07	96.9			
301–500	17	2665	6250	0.422	0.360, 0.486	410.77	96.0			
501–700	9	2043	4915	0.411	0.339, 0.487	232.71	96.4			
>700	7	6591	23 109	0.352	0.307, 0.401	427.81	97.2			
Measurement and cut-off score								96.8	18.436	0.299
Anxiety and depression emotion scale ⩾40	1	307	596	0.515	0.475, 0.555	0.00	–			
CBDI-II ⩾ 13	1	154	620	0.248	0.216, 0.284	0.00	–			
CESD-10 > 20	1	136	507	0.268	0.232, 0.309	0.00	–			
CESD-10 ⩾ 10	2	158	412	0.390	0.323, 0.462	3.21	29.9			
CESD-12 > 11	1	508	1352	0.376	0.350, 0.402	0.00	–			
CESD-12 ⩾ 9	3	755	1647	0.476	0.387, 0.565	38.21	92.4			
CESD-20 ⩾ 16	19	3966	8425	0.482	0.427, 0.537	344.91	95.8			
CESD-20 ⩾ 22	1	70	184	0.429	0.360 0.502	0.00	–			
DASS > 6.03	1	163	420	0.389	0.343, 0.436	0.00	–			
DASS ⩾ 10	1	109	225	0.484	0.420, 0.550	0.00	–			
Depression subscale of SCL-90 ⩾ 2	1	16	50	0.320	0.206, 0.460	0.00	–			
DSRSC ⩾ 15	2	4459	17 549	0.2803	0.228, 0.339	71.81	97.2			
HADS ⩾ 8	2	230	595	0.389	0.342, 0.438	2.91	29.7			
MOS-HIV	1	76	420	0.181	0.147, 0.221	0.00	–			
PHQ-9 ⩾ 10	1	132	321	0.411	0.359, 0.466	0.00	–			
SDS ⩾ 40	6	830	2207	0.398	0.298, 0.508	89.56	95.5			
SDS ⩾ 42	8	851	2001	0.477	0.294, 0.667	246.76	98.1			
Overall	52	12 887	37 376	0.432	0.389, 0.475	2596.90	96.6			

N, not reported; NGO, non-governmental organisation; RDS, respondent-driven sampling; VCT, HIV voluntary counselling and testing; MSM, men who have sex with Men; HNMSM, HIV-negative MSM; UnMSM, serostatus-unknown MSM; HIVMSM, HIV-positive MSM; YSMM, young sexual minority male; MB, money boy; CBDI-II, Chinese version of Beck Depression Inventory; CESD, Center for Epidemiological Studies Depression Scale; DASS: Depression Anxiety and Stress scale; SCL-90: Symptom Checklist 90; DSRSC, Depression Self-Rating Scale for Children; PHQ, Patient Health Questionnaire; SDS, Self-Rating Depression scale.

**Fig. 2.**
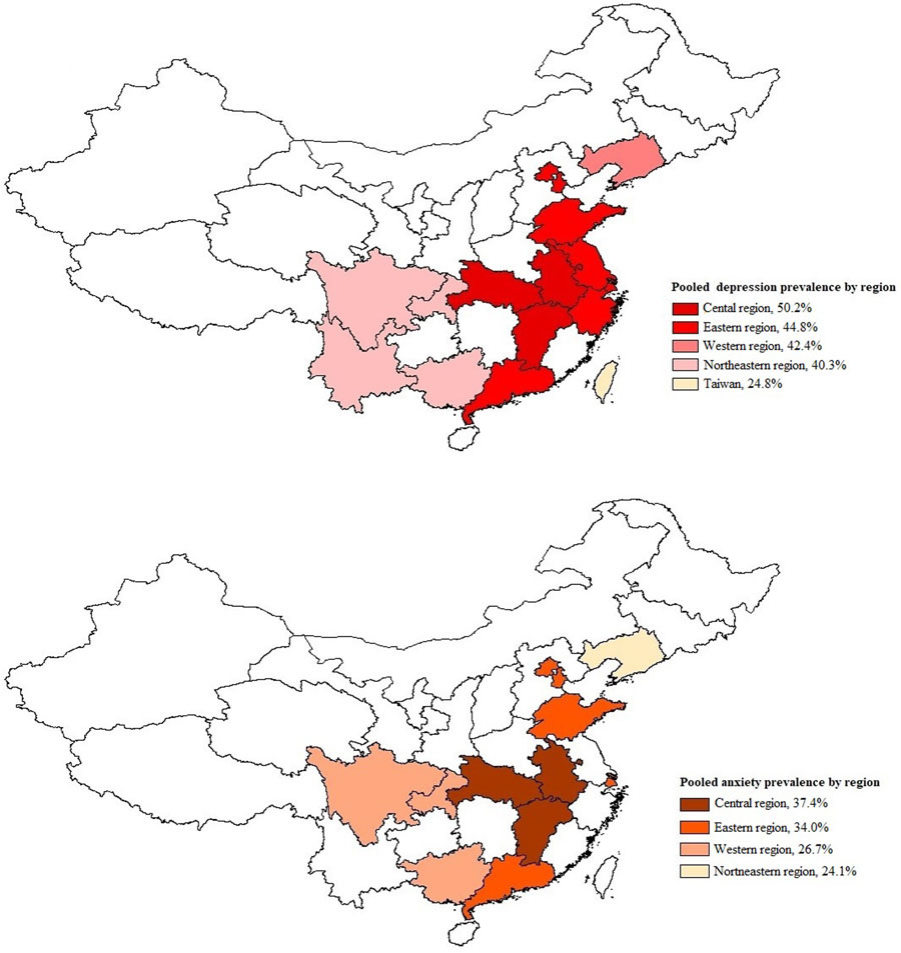
Pooled prevalence by region.

Subgroup differences were found in different study regions, survey years, sampling methods, study populations and assessment methods (*p* < 0.001). The estimated prevalence of depressive symptoms was higher in the central region (including Anhui and Jiangxi) in this study (50.2%; 95% CI, 41.2–59.1; Fig. 2). All studies conducted between 2004 and 2007 also had a higher prevalence of depressive symptoms than other sampling periods (59.0%; 95% CI, 53.7–64.0). The pooled prevalence of depressive symptoms varied according to the sampling methods. All studies using venue-based (i.e. sauna, bar; 62.3%; 95% CI, 54.6–69.4) and online (63.9%; 95% CI, 58.2–69.2) recruitment reported a higher prevalence of depressive symptoms than other recruitment methods. In addition, all studies involving HIV-positive MSM (50.5%; 95% CI, 40.7–60.2) and MB (63.1%; 95% CI, 58.0–68.0) reported a higher prevalence of depressive symptoms, while those involving HIV-negative or serostatus-unknown MSM reported a lower prevalence of depressive symptoms, ranging from 29.2 to 32.3%. The prevalence of depressive symptoms also varied with the different measurement and cut-off scores. All studies adopting a CESD-20 cut-off score of 16 or above (48.2%; 95% CI, 42.7–53.7) and an SDS cut-off score of 42 or above (47.7%; 95% CI, 29.4–66.7) reported a similar prevalence. In all univariate meta-regression analyses, only the study population could explain the heterogeneity of the estimates of the prevalence of depressive symptoms (*p* = 0.007). Publication bias was found in the pooled prevalence analysis (*p* < 0.001 using Egger's test).

### Anxiety symptoms

The pooled prevalence of anxiety symptoms was 32.2% (3187/10 531 individuals; 95% CI, 28.3–36.6; Table 2). There was substantial heterogeneity between the included studies (*I*^2^ = 95.1%). Sensitivity analysis showed that there was no significant effect of individual studies on the overall prevalence of anxiety symptoms (*p* < 0.05). In the subgroup analysis, heterogeneity was found to be reduced for the 2004–2007 period (*I*^2^ = 0.0%), in studies using venue-based sampling (*I*^2^ = 0.0%), in the YSMM population (*I*^2^ = 71.0%), for a sample size <100 (*I*^2^ = 38.9%) and in studies using a HADS cut-off score of 8 or above (*I*^2^ = 62.0%).

**Table 2.** Estimated anxiety prevalence among MSM in China

	Subgroup analysis			Meta regression			
Subgroup	No. of studies	No. of depression	Sample size	Estimated rate	95% CI	*Q*	*I*^2^ (%)	*I*^2^ (%)	*Q*_m_	*p*	*R*^2^ (%)
Study region								94.9	3.563	0.468	0.0
Central	5	332	899	0.374	0.310, 0.452	18.50	78.4				
Eastern	14	1383	4121	0.340	0.283, 0.409	237.57	94.5				
Multiple	3	718	2658	0.291	0.226, 0.375	27.07	92.6				
Northeastern	2	299	1243	0.241	0.072, 0.805	135.65	92.6				
Western	3	455	1610	0.267	0.216, 0.331	12.13	83.5				
Survey year								95.0	3.861	0.425	0.0
2004–2007	2	130	283	0.459	0.405, 0.521	0.03	0.0				
2008–2011	2	299	1243	0.241	0.072, 0.805	135.65	99.3				
2012–2015	18	2253	7629	0.313	0.274, 0.357	235.5	92.8				
2016–2019	2	238	623	0.370	0.154, 0.889	67.45	98.5				
N	3	267	753	0.356	0.324, 0.392	1.74	0.0				
Sampling methods								93.7	11.703	**0.039**	14.6
Multiple methods	2	736	2663	0.286	0.230, 0.355	12.06	91.7				
RDS	3	249	729	0.265	0.150, 0.466	112.32	98.2				
Snowball	8	1083	4516	0.256	0.203, 0.323	80.42	91.3				
VCT clinic	10	1266	3496	0.364	0.299, 0.442	125.67	92.8				
Venue-based	2	190	372	0.459	0.405, 0.521	0.03	0.0				
N	2	265	595	0.451	0.390, 0.521	2.63	62.0				
Study population								94.0	7.537	0.110	11.2
HIVMSM	10	1081	2793	0.387	0.328, 0.457	112.32	92.0				
HN/UnMSM	1	463	1809	0.256	0.237, 0.277	0.00	–				
MB	1	54	116	0.466	0.383, 0.566	0.00	–				
MSM	11	1335	4935	0.283	0.226, 0.353	241.41	95.9				
YSMM	4	254	878	0.279	0.220, 0.354	10.36	71.0				
Sample size								91.8	21.225	**<0.001**	39.2
<100	4	80	243	0.330	0.260, 0.417	4.91	38.9				
100–300	7	565	1229	0.445	0.384, 0.515	33.33	82.0				
301–500	9	1140	3335	0.336	0.292, 0.387	72.17	88.9				
501–700	4	557	2254	0.230	0.157, 0.37	81.5	96.3				
>700	3	841	3470	0.222	0.151, 0.326	75.04	97.3				
Measurement and Cut-off Score								95.7	5.719	0.573	0.0
Anxiety and depression emotion	1	217	596	0.364	0.327, 0.405	0.00	–				
Anxiety subscale of SCL-90 ⩾ 2	1	13	50	0.260	0.163, 0.415	0.00	–				
DASS > 5.26	1	162	420	0.386	0.342, 0.435	0.00	–				
GAD-7 ⩾ 10	3	257	1175	0.225	0.132, 0.382	49.4	96.0				
GAD-7 ⩾ 5	1	32	76	0.421	0.324, 0.548	0.00	–				
HADS ⩾ 8	2	265	595	0.451	0.390, 0.521	2.63	62.0				
MOS-HIV	1	143	420	0.341	0.298, 0.389	0.00	–				
SAS ⩾ 40	17	2098	7199	0.319	0.267, 0.381	393.43	95.9				
Overall	27	3187	10 531	0.322	0.283, 0.366	529.45	95.1				

GAD-7, General Anxiety Disorder-7 item; SAS, Self-Rating Anxiety Scale.

Subgroup analysis showed that the pooled prevalence of anxiety symptoms varied by survey year, sampling method, sample size, study population and measurement (*p* < 0.05). The estimated prevalence of anxiety symptoms was higher in the central region of China (37.4%; 95% CI, 31.0–45.2; Fig. 2). All studies conducted between 2004 and 2007 also had a higher prevalence of anxiety symptoms than other sampling periods (45.9%; 95% CI, 40.5–52.1). All studies using venue-based recruitment (45.9%; 95% CI, 40.5–52.1) reported a higher prevalence of anxiety symptoms than other recruitment methods. Similarly, all studies involving HIV-positive MSM (38.7%; 95% CI, 32.8–45.7) and MB (46.6%; 95% CI, 38.3–56.6) reported a higher prevalence of anxiety symptoms than those involving HIV-negative or serostatus-unknown MSM (25.6–29.4%). Studies with a smaller sample reported a higher prevalence of anxiety symptoms than those with a larger sample (33.0–44.5% *v*. 22.2–23.0%). The prevalence of anxiety symptoms measured using the SAS with a cut-off score of 40 or above (31.9%; 95% CI, 26.7–38.1), used in most studies to identify significant anxiety symptoms, was similar to the overall prevalence. In all univariate meta-regression analyses, the sampling methods (*p* = 0.039, *R*^2^ = 14.6%) and the sample size (*p* < 0.001, *R*^2^ = 39.2%) partially explained the heterogeneity of the estimates of the prevalence of anxiety symptoms. Egger's test showed that there was no significant publication bias in the pooled prevalence analysis (*p* = 0.353).

### Suicide

As the number of studies on suicide (including suicide ideation, suicide plans and suicide attempts) was small and the recall time used in these studies was different, we reported the combined prevalence of suicidal ideation, suicide plans and suicide attempts at different recall times (Table 3). Among suicidal ideation studies, the most commonly used recall time was ‘over the past week’ (four studies), ‘over the past year’ (seven studies) and ‘lifetime’ (six studies), with a combined prevalence of 20.3% (95% CI, 12.6–32.7), 18.1% (95% CI, 13.0–25.1) and 24.0% (95% CI, 20.1–28.6), respectively. There was significant heterogeneity in each subgroup (*I*^2^ = 90.0–97.3%). Four studies with high heterogeneity (*I*^2^ = 85.6%) reported a combined prevalence of suicide plans over the past year of 5.5% (95% CI, 20.1–28.6). Among the studies reporting suicide attempts, the most commonly used recall time was ‘over the past year’ (six studies) and ‘lifetime’ (four studies), with a combined prevalence of 4.2% (95% CI, 2.2–6.3) and 8.6% (95% CI, 3.8–13.3), respectively. There was significant heterogeneity in each subgroup (*I*^2^ = 92.0–96.6%).

**Table 3.** Subgroup analysis of suicidality according to different recall times

Recall time	No. of studies	No. of depression	Sample size	Estimated rate	95% CI	*Q*	*I*^2^ (%)
Suicidal ideation							
7 days	4	242	1142	0.203	0.126, 0.327	50.23	94.0
1 month	1	42	410	0.102	0.077, 0.136	0.00	–
12 months	7	1365	6059	0.181	0.130, 0.251	218.91	97.3
6 months	2	75	385	0.169	0.050, 0.571	24.57	95.9
Lifetime	6	1082	4661	0.240	0.201, 0.286	49.94	90.0
Since HIV diagnosis	1	108	225	0.480	0.419, 0.550	0.00	–
N	2	502	2152	0.271	0.166, 0.442	33.31	97.0
Overall	23	3416	15 034	0.212	0.183, 0.245	506.96	95.7
Suicide plans							
1 month	2	22	638	0.034	0.020, 0.048	0.25	0.0
12 months	4	147	2581	0.055	0.032, 0.078	20.85	85.6
6 months	1	9	201	0.045	0.016, 0.073	0.00	–
Lifetime	2	223	1851	0.115	0.087, 0.142	2.43	58.9
Overall	9	401	5271	0.062	0.039, 0.086	105.46	92.4
Suicide attempts							
7 days	2	31	279	0.091	0.000, 0.235	22.32	95.5
1 month	1	26	228	0.114	0.073, 0.155	0.00	–
6 months	2	17	385	0.042	0.022, 0.062	0.85	0.0
12 months	6	985	20 130	0.042	0.022, 0.063	145.90	96.6
Lifetime	4	157	1661	0.086	0.038, 0.133	37.61	92.0
Since HIV diagnosis	1	6	225	0.027	0.006, 0.048	0.00	–
N	4	607	5028	0.027	0.006, 0.048	0.00	–
Overall	19	1829	27 936	0.073	0.056, 0.090	510.60	96.3

## Discussion

### Main findings

The above study showed a high prevalence of depressive symptoms, anxiety symptoms and suicide among Chinese MSM compared with the general population. The meta-analysis revealed that 43.2% (ranging from 18.1 to 70.9%) of all MSM screened positive for depressive symptoms, which was more than double the prevalence in the general Chinese population (16.6%) (Huang *et al*., 2019) and higher than MSM in other countries (10.6–36.7%) (Mills *et al*., 2004; Parker *et al*., 2015; Hylton *et al*., 2017; Semple *et al*., 2017; Korhonen *et al*., 2018). Sexual minorities in China are more hidden than in other countries, with only 5% revealing their sexual identity, leaving the public with little awareness and very skeptical about this population. Moreover, traditional family values are deeply rooted in Chinese society and the majority of sexual minorities are excluded from the family. They also face increased difficulties in accessing medical and social services because of their sexual identity. Coupled with the high prevalence of HIV among MSM in China, MSM suffer from a double stigma. All of these factors contribute to the higher prevalence of psychological problems among MSM in China than in other countries (UNDP, 2016; Sun *et al*., 2020).

The summary prevalence of anxiety symptoms among all MSM was 32.2% (12.2–57.6%), with a higher prevalence among MB (46.6%) and HIV-positive MSM (38.7%). In addition, the prevalence of depression and anxiety symptoms was higher among MSM living in the central region and during the 2004–2007 period. The summary prevalence varied by assessment method, with the most frequently used methods yielding a similar prevalence. Moreover, Chinese MSM reported 18.1 and 24.0% of suicidal ideation in the past year and in their lifetime, 5.5 and 11.5% of suicide plans in the past year and in their lifetime and 4.2 and 8.6% of suicide attempts in the past year and in their lifetime. In comparison, only 3.9 and 0.80% of the general population reported suicide ideation and suicide attempts in their lifetime (Cao *et al*., 2015). However, given the methodological limitations of the included studies, these results should be interpreted with caution.

### Limitations of included studies

The included studies had several methodological limitations. First, all included studies used different methodologies to investigate sexual identities, including self-reported sexual orientation or sexual attraction, self-reported same-sex sexual encounters, or both. Few studies distinguished between gay, bisexual and homosexual MSM during screening. However, men's mental health status may be influenced by their internal sexual identification, and previous studies have suggested that bisexual men (defined by identity, behaviour or attraction) are more at risk for mental health problems than gay men (Bostwick *et al*., 2010; Conron *et al*., 2010). Therefore, additional studies distinguishing between sexual identities are needed.

Second, considering the identity concealment of MSM, it is difficult to obtain a large sample. Some of the included studies used a small sample, which could lead to a biased estimated prevalence. In addition, there were few cross-regional or national studies, and most studies were conducted in developed urban cities. Therefore, cross-regional studies and studies focusing on MSM in the central, western and northeast regions of China are needed to contribute to a more comprehensive understanding of mental health problems among Chinese MSM and develop interventions in the context of regional differences.

Finally, all included studies used different measures for depressive symptoms, anxiety symptoms and suicide. Although these studies used standardised scales, such as the CES-D, the SDS or the SAS, they did not choose the same cut-off score, which greatly affected their final results. In addition, most included studies used a single item to investigate suicide ideation, suicide plans or suicide attempts, with inconsistent statements, and only some studies explicitly defined suicidal ideation, suicide plans or suicide attempts. Unstandardised screening tools and inconsistent definitions of suicide may have led to inaccurate results. To the best of our knowledge, the definition of suicide and related terms vary across cultures and societies, thus there is no universal scale for suicide screening (Xiao and Xu, 2005). However, we encourage researchers to use standardised scales that have been culturally validated instead of a single item.

### Strengths and limitations of this study

Our review involved an extensive literature search with no time restrictions and quantitatively summarised the prevalence of depressive symptoms, anxiety symptoms and suicide among Chinese MSM. When interpreting these findings, it is important to recognise that the data synthesised in this study came from self-reported measures of depressive symptoms, anxiety symptoms and suicide, which were strongly influenced by the sensitivity and specificity of the screening instruments. Meanwhile, the cut-off points set to screen for depressive and anxiety symptoms and the recall time used to estimate suicidal behaviours varied across different studies, leading to significant heterogeneity in overall pooled prevalence. In addition, the factors that can be sources of heterogeneity, such as different sexual orientations or the severity of depressive symptoms or anxiety symptoms, could not be extracted for analysis, leaving substantial heterogeneity between studies largely unexplained by the variables studied. Finally, the studies mainly used a cross-sectional design and only the baseline data were extracted from the cohort studies, whereas longitudinal studies on the change in prevalence were lacking. Therefore, the results of longitudinal studies should be followed in future studies.

### Implications for future research

First, further studies should clarify participants' sexual orientation and self-identity during screening, which can help determine the potential impact of internal sexual identification on their mental health status. Second, national studies using probability-based samples are needed to establish more reliable estimates of the prevalence of different psychological problems among MSM. Third, longitudinal studies are also needed to establish the causal pathway between mental health problems and related factors, which are greatly warranted for intervention development. Fourth, for the measurement of depression and anxiety, attention should be paid to the standardised use of the scale and the cut-off score. Fifth, the existing studies basically measure depressive or anxiety symptoms rather than clinically significant depression and anxiety disorders. Further studies should focus on the incidence of depression or anxiety disorders and identify the burden of untreated mental disorders among MSM, so as to provide reference for the allocation of mental health services and medical resources for this population. Sixth, this review highlighted high prevalence of mental health problems of Chinese MSM population and their needs for mental health interventions and services. More intervention studies are needed to find ways to address their poor mental health. Rigorous assessments of the feasibility, effectiveness and sustainability of mental health service and interventions for MSM are also warranted to guide future policies and practices.

## Conclusions

Depressive symptoms, anxiety symptoms and suicide are common mental health problems among Chinese MSM. Additional attention is needed to provide mental health services and to identify those at risk of mental health problems and those requiring treatment. Efforts are needed to develop interventions to prevent and alleviate these mental health problems in this population.

## Supplementary material

For supplementary material accompanying this paper visit http://dx.doi.org/10.1017/S2045796020000487. click here to view supplementary material

## Data

The data sets used and analysed in the current study are available from the corresponding author on reasonable request.
