# Supplementary material for: Prevalence of depression, anxiety and suicide among men who have sex with men in China: a systematic review and meta-analysis
Source: Epidemiol Psychiatr Sci. 2020 Jun 15;29:e136. doi: 10.1017/S2045796020000487 (PMC7303796; doi:10.1017/S2045796020000487)

**Figure 1. Forest plot of pooled depression prevalence**


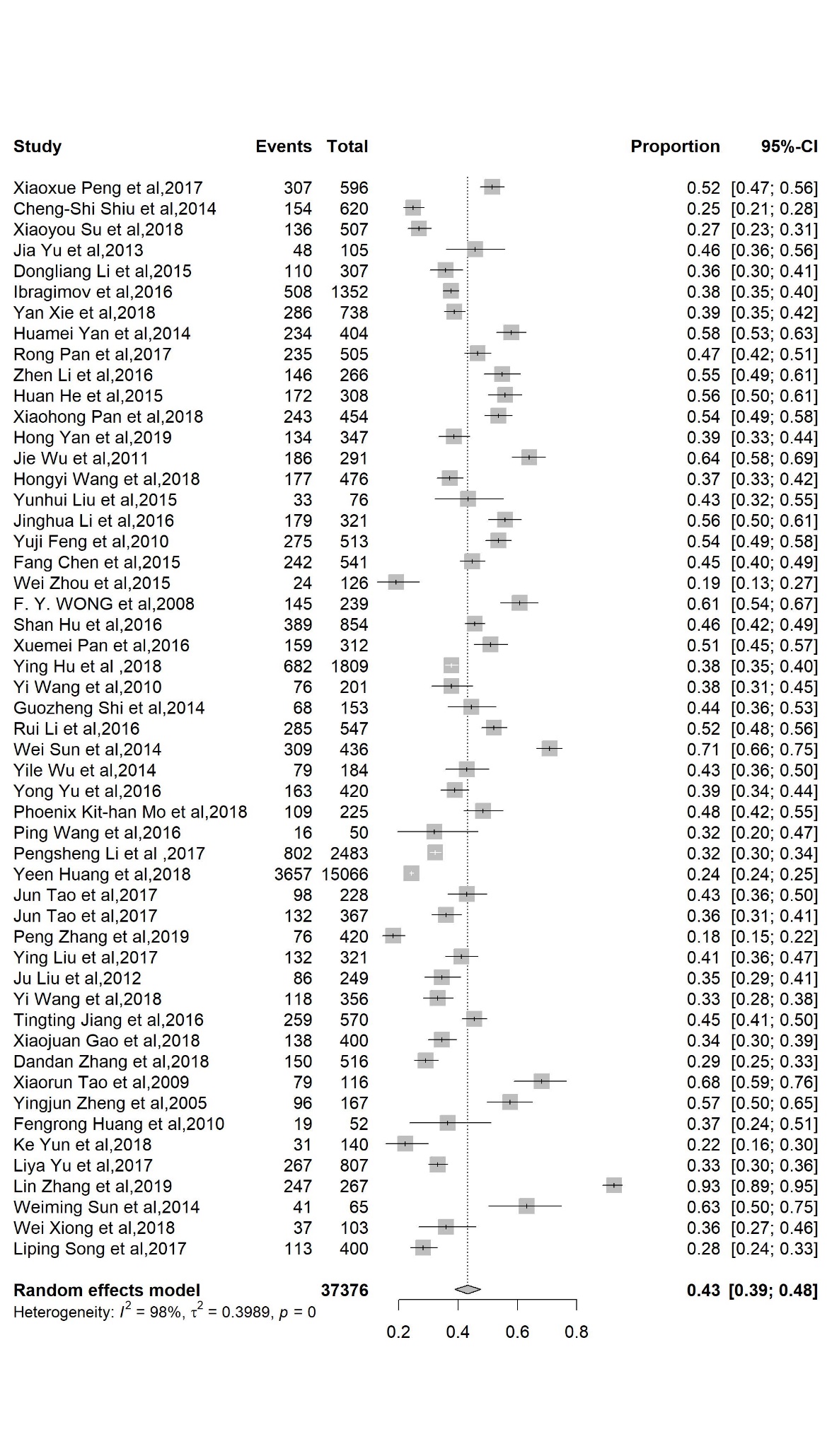


**Figure2. Forest plot of pooled anxiety prevalence**


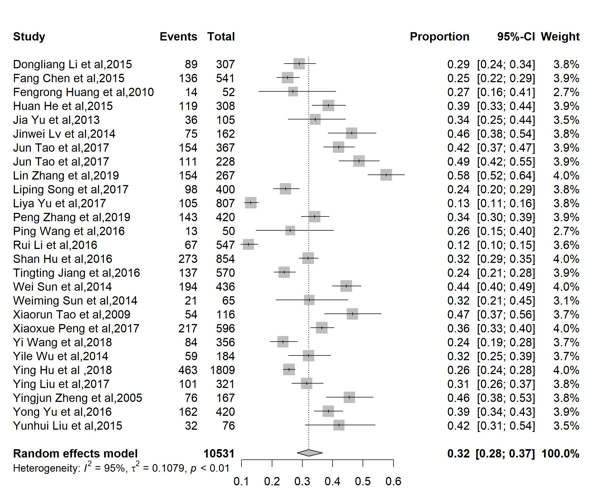


**Figure 3. Forest plot of sensitivity analysis for pooled depression prevalence**


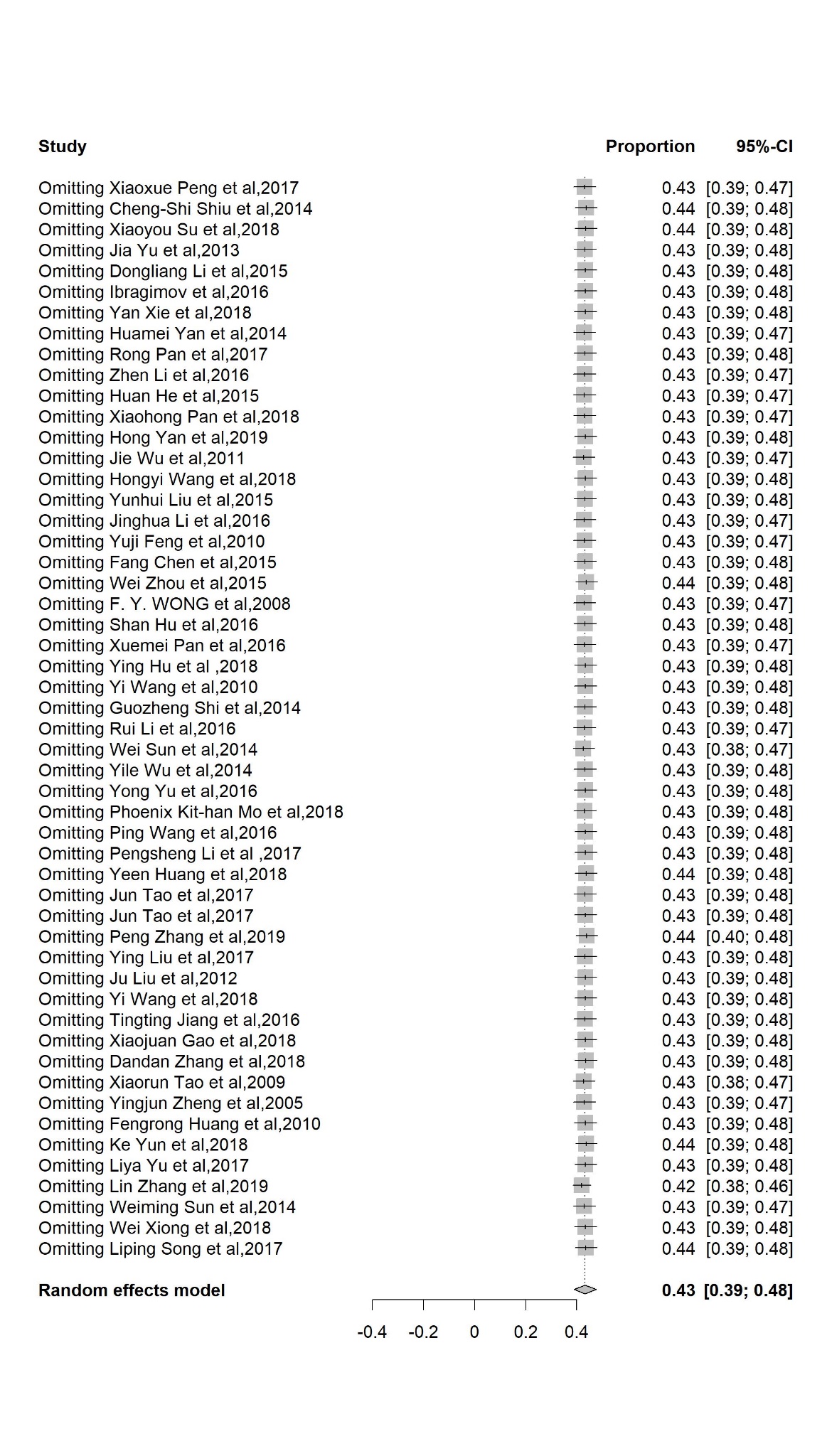


**Figure 4. Forest plot of sensitivity analysis for pooled anxiety prevalence**


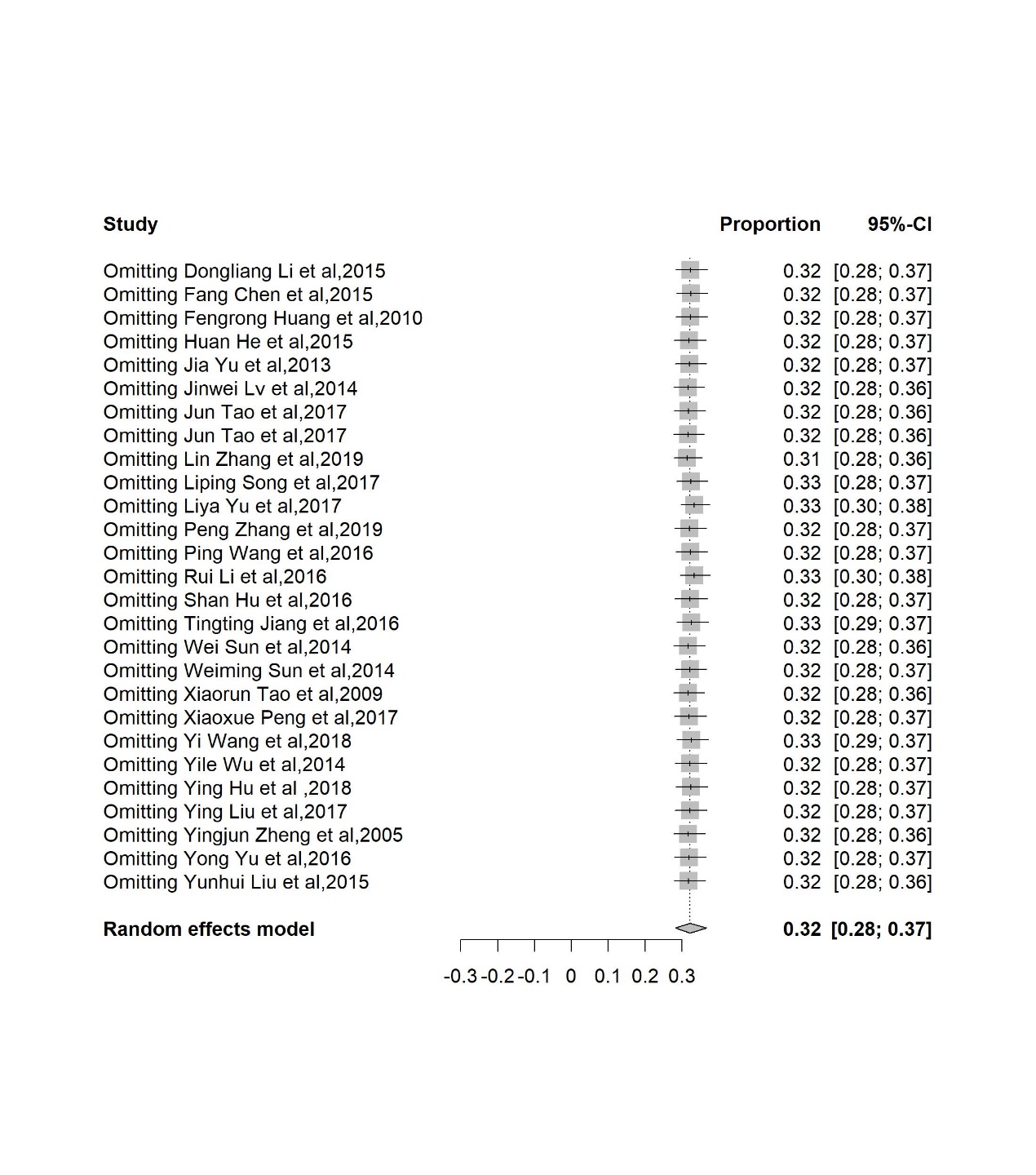

Supplement: Supplementary file 1 [file S2045796020000487sup001.zip › S2045796020000487sup001.docx]
