# Supplementary material for: Prevalence of depression, anxiety and suicide among men who have sex with men in China: a systematic review and meta-analysis
Source: Epidemiol Psychiatr Sci. 2020 Jun 15;29:e136. doi: 10.1017/S2045796020000487 (PMC7303796; doi:10.1017/S2045796020000487)

**Figure 1. Funnel plot of pooled depression prevalence**


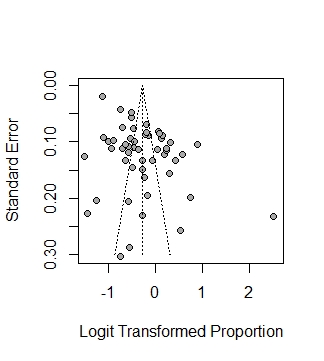


**Figure 2. Funnel plot of pooled anxiety prevalence**


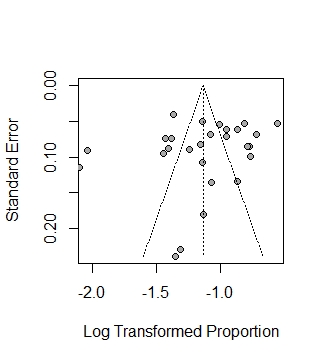

Supplement: Supplementary file 1 [file S2045796020000487sup001.zip › S2045796020000487sup002.docx]
