# Supplementary material for: Prevalence of depression, anxiety and suicide among men who have sex with men in China: a systematic review and meta-analysis
Source: Epidemiol Psychiatr Sci. 2020 Jun 15;29:e136. doi: 10.1017/S2045796020000487 (PMC7303796; doi:10.1017/S2045796020000487)
Supplement: Supplementary file 1 [file S2045796020000487sup001.zip › S2045796020000487sup003.docx]

| **条目** | **Items in English** |
| --- | --- |
| 研究目的是否明确，立题依据是否充分？ | Was the purpose of the study clear and the basis of the topic sufficient? |
| 是否详细描述研究对象及研究场所？ | Were the study subjects and the setting described in detail? |
| 是否清楚描述样本的纳入和排除标准？ | Were inclusion and exclusion criteria for study subjects clearly described? |
| 研究人群如何选择？抽样方法？是否随机？ | Was the sample frame appropriate to address the target population? |
| 资料收集的工具是否具有信度和效度？ | Were valid methods used for the identification of the condition? |
| 是否对保证资料的真实性采取了恰当的措施？ | Were appropriate measures taken to ensure the authenticity of the information? |
| 是否考虑到伦理问题？ | Were ethical issues considered? |
| 统计方法是否正确？ | Was there appropriate statistical analysis? |
| 对结果的陈述和分析是否恰当、准确？ | Was the presentation and analysis of the results appropriate and accurate? |
| 是否清晰阐述研究的价值？ | Was the value of the research clearly stated? |

Table S2. The Joanna Briggs Institute Critical Appraisal tools for use in JBI Systematic Reviews Checklist for Prevalence Studies (JBI checklist) used in this study
