# Supplementary material for: Prevalence of depression, anxiety and suicide among men who have sex with men in China: a systematic review and meta-analysis
Source: Epidemiol Psychiatr Sci. 2020 Jun 15;29:e136. doi: 10.1017/S2045796020000487 (PMC7303796; doi:10.1017/S2045796020000487)
Supplement: Supplementary file 1 [file S2045796020000487sup001.zip › S2045796020000487sup005.docx]

**Quality score of included studies**

| Study | Quality score |
| --- | --- |
| Cheng-Shi Shiu et al,^1^2014 | 13 |
| Dandan Zhang et al,^2^2018 | 12 |
| Dongliang Li et al,^3^2015 | 17 |
| F. Y. WONG et al,^4^2008 | 16 |
| Fang Chen et al,^5^2015 | 10 |
| Fengrong Huang et al,^6^2010 | 10 |
| Guanzhi Chen et al,^7^2012 | 16 |
| Guozheng Shi et al,^8^2014 | 10 |
| Hong Yan et al,^9^2019 | 15 |
| Hongbo Zhang et al,^10^2007 | 10 |
| Hongquan Chen et al,^11^2011 | 11 |
| Hongquan Chen et al,^12^2015 | 9 |
| Hongyi Wang et al,^13^2018 | 17 |
| Huamei Yan et al,^14^2014 | 9 |
| Huan He et al, ^15^2015 | 13 |
| Huijuan Mu et al,^16^12016 | 15 |
| Ibragimov et al,^17^2016 | 17 |
| Jia Yu et al,^18^2013 | 10 |
| Jie Wu et al,^19^2011 | 10 |
| Jing Li et al,^20^2018 | 12 |
| Jinghua Li et al,^21^2016 | 16 |
| Jinwei Lv et al,^22^2014 | 7 |
| Ju Liu et al,^23^2012 | 12 |
| Jun Tao et al,^24^2017 | 13 |
| Jun Tao et al,^25^2017 | 12 |
| Ke Yun et al,^26^2018 | 12 |
| Lijian Xue et al,^27^2011 | 6 |
| Lin Zhang et al,^28^2019 | 11 |
| Liping Song et al,^29^2017 | 12 |
| Liya Yu et al,^30^2017 | 17 |
| Peng Zhang et al,^31^2019 | 17 |
| Pengsheng Li et al,^32^2017 | 16 |
| Phoenix Kit-han Mo et al,^33^2018 | 14 |
| Ping Wang et al,^34^2016 | 8 |
| Rong Pan et al,^35^2017 | 14 |
| Rui Li et al,^36^2016 | 19 |
| Shan Hu et al,^37^2016 | 10 |
| Tingting Jiang et al,^38^2016 | 10 |
| Wei Sun et al,^39^2014 | 15 |
| Wei Xiong et al,^40^2018 | 12 |
| Wei Zhou et al,^41^2015 | 11 |
| Weiming Sun et al,^42^2014 | 11 |
| Wu Zheng et al,^43^2012 | 12 |
| Xiaohong Pan et al,^44^2018 | 16 |
| Xiaojuan Gao et al,^45^2018 | 9 |
| Xiaoerun Tao et al,^46^2009 | 7 |
| Xiaoxue Peng et al,^47^2017 | 11 |
| Xiaoyou Su et al,^48^2018 | 15 |
| Xuemei Pan et al,^49^2016 | 12 |
| Yan Xie et al,^50^2018 | 10 |
| Yang Li et al, ^51^2010 | 10 |
| Yeen Huang et al, ^52^2017 | 13 |
| Yeen Huang et al,^53^ 2018 | 15 |
| Yi Wang et al,^54^2010 | 10 |
| Yi Wang et al, ^55^2011 | 12 |
| Yi Wang et al,^56^2018 | 14 |
| Yile Wu et al,^57^2014 | 15 |
| Yilu Qin et al,^58^2017 | 11 |
| Ying Hu et al,^59^2018 | 18 |
| Ying Liu et al,^60^017 | 14 |
| Yingjun Zheng et al, ^61^2004 | 7 |
| Yong Yu et al,^62^2016 | 14 |
| Yong Yu et al, ^63^2017 | 19 |
| Yuanyuan Wang et al, ^64^2018 | 12 |
| Yuji Feng et al,^65^2010 | 16 |
| Yunhui Liu et al,^66^2015 | 7 |
| Zhen Li et al,^67^2016 | 16 |

**Reference**

1. Shiu C-S, Chen Y-C, Tseng P-C, et al. Curvilinear relationship between depression and unprotected sexual behaviors among men who have sex with men. *J Sex Med.* 2014;11(10):2466-2473.

2. Zhang D-D, Hong H, Jiang H-B. Depression in men who have sex with men in Ningbo. *Prev Med.* 2018;30(5):442-445.

3. Li D, Li C, Wang Z, Lau JTF. Prevalence and associated factors of unprotected anal intercourse with regular male sex partners among HIV negative men who have sex with men in China: a cross-sectional survey. *PloS One.* 2015;10(3):e0119977-e0119977.

4. Wong FY, Huang ZJ, He N, et al. HIV risks among gay- and non-gay-identified migrant money boys in Shanghai, China. *AIDS Care.* 2008;20(2):170-180.

5. Chen F, Lin X-J, Wang X-D, et al. Prevalence rates of depression and anxiety in HIV-infected men who have sex with men. *Chin Ment Health J.* 2015;29(4):251-257.

6. Huang FR, Huang H, Zhuang MH, et al. Investigations on social psychology and sexual behavior of 52 male homosexual adolescents in Shanghai. *Shanghai Jiao Tong Da Xue Xue Bao Yi Xue Ban.* 2010;30(5):581-584.

7. Chen G, Li Y, Zhang B, et al. Psychological characteristics in high-risk MSM in China. *BMC Public Health.* 2012;12:58.

8. Guozheng S. Study on depression status and related factors among men who have sex with men in Jiading District. *Chinese Primary Health Care.* 2014;28(4):65-67.

9. Yan H, Li X, Li J, et al. Association between perceived HIV stigma, social support, resilience, self-esteem, and depressive symptoms among HIV-positive men who have sex with men (MSM) in Nanjing, China. *AIDS Care.* 2019;31(9):1069-1076.

10. Zhang H, Zheng Y, Xu J, et al. Relationship between suicide behaviors and social character among men having sex with men in Hefei. *Chin J Public Health.* 2007;23(9):1027-1029.

11. Chen H, Zhang B, Li X. Study on high-risk behaviour and suicide associated risk factors related to HIV/AIDS among gay or bisexual men. *Chin J Epidemiol.* 2011;32(10):983-986.

12. Chen H, Li Y, Wang L, Zhang B. Causes of suicidal behaviors in men who have sex with men in China: a national questionnaire survey. *BMC Public Health.* 2015;15:91.

13. Wang H-Y, Wang N, Chu Z-X, et al. Intimate partner violence correlates with a higher HIV incidence among MSM: a 12-month prospective cohort study in Shenyang, China. *Sci Rep.* 2018;8(1):2879.

14. Yan H, Wong FY, Zheng T, et al. Social support and depressive symptoms among ‘money’ boys and general men who have sex with men in Shanghai, China. *Sex Health.* 2014;11(3):285-287.

15. He H, Zhang H, Ding F, et al. Risky sexual transmission behavior and its influencing factors among HIV-positive MSM population in Shanghai and Chengdu in China. *Chin J Epidemiol.* 2015;36(3):254-258.

16. Mu H, Li Y, Liu L, et al. Prevalence and risk factors for lifetime suicide ideation, plan and attempt in Chinese men who have sex with men. *BMC Psychiatry.* 2016;16:117.

17. Ibragimov U, Harnisch JA, Nehl EJ, et al. Estimating self-reported sex practices, drug use, depression, and intimate partner violence among MSM in China: A comparison of three recruitment methods. *AIDS Care.* 2017;29(1):125-131.

18. Yu J, Wang Z, Li C, Liu, D. Mental health status of MSM in Beijing. *Chin J AIDS STD.* 2013;19(4):241-243.

19. Wu J, Lu C, Hao Y, et al. Psychosocial status of MSM population in Guangzhou City. *Chin J Public Health.* 2011;27(4):476-478.

20. Li J, Mo PKH, Wu AMS, Lau JTF. Roles of self-stigma, social support, and positive and negative affects as determinants of depressive symptoms among HIV infected men who have sex with men in China. *AIDS Behav.* 2017;21(1):261-273.

21. Lv J, Cheng G, Cheng Z. Investigation on anxiety and intervention effect among men who have sex with men in Wuhu City. *Occup and Health.* 2014;30(13):1836-1838.

22. Zheng Y, Xu J, Zhao B, Zhang H. Psychosocial distinction of men who have sex with men. *Chin Behav Med Sci.* 2004;13(6):655-657.

23. Liu J, Gao Y-H, Liang Z-M, Li Y, Yang Y. Depressive symptoms and associated sexual behaviors among men who have sex with men in Foshan, Guangdong province. *Zhonghua Liu Xing Bing Xue Za Zhi.* 2012;33(5):483-487.

24. Tao J, Vermund SH, Lu H, et al. Impact of depression and anxiety on initiation of antiretroviral therapy among men who have sex with men with newly diagnosed HIV infections in China. *AIDS Patient Care STDS.* 2017;31(2):96-104.

25. Tao J, Qian H-Z, Kipp AM, et al. Effects of depression and anxiety on antiretroviral therapy adherence among newly diagnosed HIV-infected Chinese MSM. *AIDS.* 2017;31(3):401-406.

26. Yun K, Chu Z, Hu Q, et al. Association between psychological disorders, depression and the CD4 level of HIV negative MSM in Shenyang. *Chin J AIDS STD.* 2018;24(11):1118-1123.

27. Xue L, Xu Y, Hong Z, Shi J, Zhao X, Zhu W. An online survey of HIV related knowledge and high risk behaviors, social pressure and their influencing factors among MSM. *Chin J AIDS STD.* 2011;17(3):324-327.

28. Zhang L, Zhou Y, et al. Anxiety states of homosexual AIDS men who have heterosexual marriage. *Shanghai Journal of Preventive Medicine.* 2019;31(4):294-300.

29. Song L, Zhang Y, Lan G, et al. The impacting factor of peer education among MSM. *Chin J Dis Control Prev.* 2017;21(11):1132-1135.

30. Yu L, Li Y, Liu L, et al. Association of recent gay-related stressful events and emotional distress with suicidal behaviors over 12 months in Chinese men who have sex with men. *Asia Pac Psychiatry.* 2018;10(1):1758-5872.

31. Zhang P, Gao J, Wang Y, Sun Q, Sun X. Effect of chronic disease self-management program on the quality of life of HIV-infected men who have sex with men: an empirical study in Shanghai, China. *Int J Health Plann Manage.* 2019;34:1055-1064.

32. Li P, Huang Y, Guo L, et al. Is sexual minority status associated with poor sleep quality among adolescents? Analysis of a national cross-sectional survey in Chinese adolescents. *BMJ Open.* 2017;7(12):e017067.

33. Mo PK-H, Lau JT-F, Wu X. Relationship between illness representations and mental health among HIV-positive men who have sex with men. *AIDS Care.* 2018;30(10):1246-1251.

34. Wang P, Liu Z, Guo Y, Zhang X. Association between mental health and family environment among young male students who have sex with men. *Chin J Sch Health.* 2016;37(4):523-526.

35. Pan R, Kun C, Zheng H, et al. Depressive symptoms and related factors among HIV-positive men who have sex with men in Shanghai. *Fudan Univ J Medical Sci.* 2017;44:430-434.

36. Li R, Cai Y, Wang Y, Gan F, Shi R. Psychological pathway to suicidal ideation among men who have sex with men in Shanghai, China: a structural equation model. *J Psychiatr Res.* 2016;83:203-210.

37. Hu S, Zhong X-N, Wen X-Y, et al. Characteristics of anxiety and depression symptoms among gays and bisexual men with HIV negative. *Chinese Mental Health Journal.* 2016;30(3):213-219.

38. Jiang T, Wang H, Zhou X, Ma Q. Depressive, anxiety symptoms and related influential factors among men who have sex with men in Zhejiang. *Chin J AIDS STD.* 2016;22:357-360.

39. Sun W, Wu M, Qu P, Lu C, Wang L. Psychological well-being of people living with HIV/AIDS under the new epidemic characteristics in China and the risk factors: a population-based study. *Int J Infect Dis.* 2014;28:e147-e152.

40. Xiong W, Zhang R, Liu Y, Yin L. Depression of gay college students in Chengdu and its influencing factors. *Chin J Sch Health.* 2018;39(8):1239-1241.

41. Wang Y, Xu J, Zhang G, Yang H, Fan J. Depressive symptoms and related factors in MSM. *Chinese Mental Health Journal.* 2010;24(5):366-367,369.

42. Sun W, Yuanye F, Xu D, Cheng X, Zou Q, Lou Q. The anxiety and depression of male homosexual with HIV/AIDS in Nanchang City: current situation and related factors. *Chin J Dis Control Prev.* 2014;18:1143-1146.

43. Zheng W, Wu C, Zhang B, Pi Q, Liu W. Analysis on influencing factors of suicide behavior among “MSM” in Wuhan. *J Public Health Prev Med.* 2012;23(1):51-55.

44. Pan X, Li R, Ma Q, et al. Sexual risk behaviour, sexual victimisation, substance use and other factors related to depression in men who have sex with men in Wenzhou, China: a cross-sectional study. *BMJ Open.* 2018;8(4)-2016-013512.

45. Gao X, Li Y, et al. Depression and related influential factors among men who have sex with men in Kunming. *Chin J AIDS STD.* 2018;24:170-173.

46. Tao X, Zheng W, Liu J, et al. Mental status of money boys in Shandong province. *Chin J Public Health.* 2009;25(9):1092-1093.

47. Peng X, Qi J, Chen M. State of anxiety and depression and related influencing factors among men who have sex with men in Shenzhen. *Chin J AIDS STD.* 2017;7:630-633.39.

48. Su X, Zhou AN, Li J, et al. Depression, loneliness, and sexual risk-taking among HIV-negative/unknown men who have sex with men in China. *Arch Sex Behav.* 2018;47(7):1959-1968.

49. Pan X, Zheng W, Huang J, Deng Y, Chen Z. Investigation of current status of depression, quality of life and influencing factors among men who have sex with men in Lianzhou. *Jiangsu J Prev Med.* 2016;27(1):44-47.

50. Xie Y, Yang Y. Analysis on depressive symptoms and related factors of men who have sex with men attending HIV voluntary counseling and testing clinics in Shanghai. *Chinese Journal of Disease Control and Prevention.* 2018;22(12):1248-1251.

51. Li Y, Li X, Zang Y, Wang L, Chen G, Yu Z. Behavioral characteristics of men who have sex with men with sadomasochism associated with bleeding. *Chin J Epidemiol.* 2010;31(2):142-145.

52. Huang Y, Li P, Guo L, et al. Sexual minority status and suicidal behaviour among Chinese adolescents: a nationally representative cross-sectional study. *BMJ Open.* 2018;8(8):e020969.

53. Huang Y, Li P, Lai Z, et al. Chinese sexual minority male adolescents’ suicidality and body mass index. *Int J Environ Res Public Health.* 2018;15(11):2558.

54. Wang Y, Xu J, Zhang G, Yang H, Fan J. Relationship of anxiety and depressive symptoms with social behavior among young men who have sex with men. *South China J Prev Med.* 2018;44(5):401-405.

55. Wang Y, Xu J, Zhang G, Yang H, Fan J. Logistic analysis for relatively risky factors of suicidal behaviors of MSM. *Journal of Modern Preventive Medicine.* 2011;38(12):2220-2223.

56. Wang Y, Zhou W, Fan J, et al. Relationship of anxiety and depressive symptoms with social behavior among young men who have sex with men. *South China J Prev Med.* 2018;44(5):401-405.

57. Wu Y-L, Yang H-Y, Wang J, et al. Prevalence of suicidal ideation and associated factors among HIV-positive MSM in Anhui, China. *Int J STD AIDS.* 2015;26(7):496-503.

58. Qin Y, Tang W, Nowacki A, et al. Benefits and potential harms of human immunodeficiency virus self-testing among men who have sex with men in China: an implementation perspective. *Sex Transm Dis.* 2017;44(4):233-238.

59. Hu Y, Zhong X-N, Peng B, et al. Comparison of depression and anxiety between HIV-negative men who have sex with men and women (MSMW) and men who have sex with men only (MSMO): a cross-sectional study in Western China. *BMJ Open.* 2019;9(1):e023498-e023498.

60. Liu Y, Niu L, et al. Emotional problems among newly diagnosed HIV-positive men with homosexual sex behaviors. *Chinese Mental Health Journal*. 2017;31(6):471-477.

61. Zheng Y, Xu J, Zhao B, Zhang H. Psychosocial distinction of men who have sex with men. *Chin Behav Med Sci.* 2004;13(6):655-657.

62. Yu Y. Health and life satisfaction for Chinese gay men in Guangzhou, China. *J Cent South Univ.* 2017;42(12):1407-1416.

63. Yu Y, Li Y, Wang P. Gay men’s gender roles and mental health in Guangzhou. *Chin J Health Psychol.* 2016;24(11):1618-1621,1622.

64. Wang Y-Y, Dong M, Zhang Q, et al. Suicidality and clinical correlates in Chinese men who have sex with men (MSM) with HIV infection. *Psychol Health Med.* 2019;24(2):137-143.

65 Feng Y, Wu Z, Detels R, et al. HIV/STD prevalence among men who have sex with men in Chengdu, China and associated risk factors for HIV infection. *J Acquir Immune Defic Syndr.* 2010;53 Suppl 1:S74-S80.

66. Liu Y, Liu X. Investigation and analysis of mental depression and anxiety of HIV/AIDS patients in MSM. *Chin J Prev Med.* 2015;16(7):578-580.

67. Li Z, Hsieh E, Morano JP, Sheng Y. Exploring HIV-related stigma among HIV-infected men who have sex with men in Beijing, China: a correlation study. *AIDS Care.* 2016;28(11):1394-1401.
