# Supplementary material for: Prevalence of depression, anxiety and suicide among men who have sex with men in China: a systematic review and meta-analysis
Source: Epidemiol Psychiatr Sci. 2020 Jun 15;29:e136. doi: 10.1017/S2045796020000487 (PMC7303796; doi:10.1017/S2045796020000487)
Supplement: Supplementary file 1 [file S2045796020000487sup001.zip › S2045796020000487sup006.docx]

**Table S1. Details of search strategy used in this study**

| **PUBMED** | (((chinese) OR china) AND ((((((((((((Male homosexuality) OR homosexuality,male) OR gay) OR same sex attract) OR queer) OR homosexual) OR bisexual) OR men who have sex with man) OR MSM))) AND ((((((depress*) OR depressive disorder*) OR depressive disorder,major) OR major depression) OR major depressive disorder) OR MDD) |
| --- | --- |
|  | ((((chinese) OR china)) AND ((((((((((((Male homosexuality) OR homosexuality,male) OR gay) OR same sex attract) OR queer) OR homosexual) OR bisexual) OR men who have sex with man) OR MSM))) AND (((anxiety) OR anxiety disorder*) OR stress disorder*) |
|  | ((((chinese) OR china)) AND ((((((((((((Male homosexuality) OR homosexuality,male) OR gay) OR same sex attract) OR queer) OR homosexual) OR bisexual) OR men who have sex with man) OR MSM))) AND (((((((((((self injurious behavio*) OR suicide) OR suicide attempt*) OR self injurious) OR self mutilation) OR self destructive) OR self harm) OR suicide ideation*) OR suicide plan*) OR suicidality) OR suicidal*) |
| **PsycoINFO** | TX ( TX ( chinese OR china ) AND TX ( male homosexuality OR homosexuality,male OR gay OR same sex attract OR queer OR homosexual OR bisexual OR men who have sex with men OR MSM ) ) AND TX ( self injurious behavio* or suicide or suicide attempt* or self injurious or self mutilation or self destructive or self harm or suicide ideation* or suicide plan* or suicidality or suicidal* ) |
|  | TX ( TX ( chinese OR china ) AND TX ( male homosexuality OR homosexuality,male OR gay OR same sex attract OR queer OR homosexual OR bisexual OR men who have sex with men OR MSM ) ) AND TX ( depress* or depressive disorder* or depressive disorder,major or major depression or major depressive disorder or MDD ) |
|  | TX ( chinese OR china ) AND TX ( male homosexuality OR homosexuality,male OR gay OR same sex attract OR queer OR homosexual OR bisexual OR men who have sex with men OR MSM ) AND TX ( anxiety disorders or anxiety or generalized anxiety disorder OR stress disorder* ) |
| **MEDLINE** | TX ( TX ( chinese OR china ) AND TX ( male homosexuality OR homosexuality,male OR gay OR same sex attract OR queer OR homosexual OR bisexual OR men who have sex with men OR MSM ) ) AND TX ( self injurious behavio* or suicide or suicide attempt* or self injurious or self mutilation or self destructive or self harm or suicide ideation* or suicide plan* or suicidality or suicidal* ) |
|  | TX ( TX ( chinese OR china ) AND TX ( male homosexuality OR homosexuality,male OR gay OR same sex attract OR queer OR homosexual OR bisexual OR men who have sex with men OR MSM ) ) AND TX ( depress* or depressive disorder* or depressive disorder,major or major depression or major depressive disorder or MDD ) |
|  | TX ( chinese OR china ) AND TX ( male homosexuality OR homosexuality,male OR gay OR same sex attract OR queer OR homosexual OR bisexual OR men who have sex with men OR MSM ) AND TX ( anxiety disorders or anxiety or generalized anxiety disorder OR stress disorder* ) |
| **Embase** | (‘china’/exp OR china OR ‘chinese’/exp OR Chinese) AND (‘men who have sex with men’/exp OR ‘men who have sex with men’ OR ‘male homosexuality’ OR ‘homosexual male’ OR ‘gay’ OR ‘bisexual’ OR ‘msm’) AND (‘suicide/exp OR’suicide attempt’ OR ‘suicidal ideation’ OR ‘suicidal behavior’ OR ‘automutilation’ OR ‘suicidality’ OR ‘suicide’) |
|  | (‘china’/exp OR china OR ‘chinese’/exp OR Chinese) AND (‘men who have sex with men’/exp OR ‘men who have sex with men’ OR ‘male homosexuality’ OR ‘homosexual male’ OR ‘gay’ OR ‘bisexual’ OR ‘msm’) AND (‘depression’/exp OR ‘depression’ OR ‘major depression’ OR ‘depressive disorder’) |
|  | (‘china’/exp OR china OR ‘chinese’/exp OR Chinese) AND (‘men who have sex with men’/exp OR ‘men who have sex with men’ OR ‘male homosexuality’ OR ‘homosexual male’ OR ‘gay’ OR ‘bisexual’ OR ‘msm’) AND (‘anxiety’/exp OR anxiety’) |
| **CNKI** | （男同）或含（男男性行为者）或含（男同性恋）或含（同志）或含（男男性关系）或含（男双性恋）并且（中国）并且（抑郁） |
|  | （男同）或含（男男性行为者）或含（男同性恋）或含（同志）或含（男男性关系）或含（男双性恋）并且（中国）并且（焦虑） |
|  | （男同）或含（男男性行为者）或含（男同性恋）或含（同志）或含（男男性关系）或含（男双性恋）并且（中国）并且（自杀） |
| **Wanfang** | （男男性行为）或（男同性恋）或（同志）或（男男性接触）与（中国）与（抑郁） |
|  | （男男性行为）或（男同性恋）或（同志）或（男男性接触）与（中国）与（焦虑） |
|  | （男男性行为）或（男同性恋）或（同志）或（男男性接触）与（中国）与（自杀） |
