# Supplementary material for: Prevalence of depression, anxiety and suicide among men who have sex with men in China: a systematic review and meta-analysis
Source: Epidemiol Psychiatr Sci. 2020 Jun 15;29:e136. doi: 10.1017/S2045796020000487 (PMC7303796; doi:10.1017/S2045796020000487)
Supplement: Supplementary file 1 [file S2045796020000487sup001.zip › S2045796020000487sup007.docx]

**Selected characteristics of the 52 studies reporting depression or depressive symptoms**

| **Study^a^** | **Study design** | **Location** | **Survey year** | **Sampling methods** | **Study population** | **Instrument and Cut-off Score** | **Sample size** | **Men,**  **No.(%)** |
| --- | --- | --- | --- | --- | --- | --- | --- | --- |
| Cheng-Shi Shiu et al,^1^2014 | Cr | Taiwan | 2011-2012 | NGO | MSM | CBDI-Ⅱ≥13 | 620 | 154(24.8) |
| Dandan Zhang et al,^2^2018 | Cr | Ningbo | N | VCT clinic | MSM | SDS≥40 | 516 | 150(29.1) |
| Dongliang Li et al,^3^2015 | Cr | Beijing,Chengdu | 2012 | NGO | HNMSM | CESD-10≥10 | 307 | 110(35.8) |
| F. Y. WONG et al,^4^2008 | Cr | Shanghai | 2006 | Multiple methods | MB | CESD-20≥16 | 239 | 145(60.7) |
| Fang Chen et al,^5^2015 | Cr | Chengdu,Chongqing,Guangzhou | 2012-2013 | Snowball | HIVMSM | CESD-20≥16 | 541 | 242(44.7) |
| Fengrong Huang et al,^6^2010 | Cr | Shanghai | N | Snowball | YSMM | SDS≥42 | 52 | 19(36.5) |
| Guozheng Shi et al,^7^2014 | Cr | Shanghai | 2012 | NGO | MSM | CESD-20≥16 | 153 | 68(44.4) |
| Hong Yan et al,^8^2019 | Cr | Nanjing | 2016 | VCT clinic | HIVMSM | CESD-20≥16 | 347 | 134(38.6) |
| Hongyi Wang et al,^9^2018 | Cohort Study | Shenyang | 2014 | Multiple methods | MSM | CESD-20≥16 | 476 | 177(37.2) |
| Huamei Yan et al,^10^2014 | Cr | Shanghai | N | RDS | MSM,MB | CESD-12≥9 | 404 | 234(57.9) |
| Huan He et al, ^11^2015 | Cr | Shanghai,Chengdu | 2013 | Snowball | HIVMSM | CESD-20≥16 | 308 | 172(55.8) |
| Ibragimov et al,^12^2016 | Cr | Shanghai | 2008-2012 | Multiple methods | MSM,MB | CESD-12 >11 | 1352 | 508(37.6) |
| Jia Yu et al,^13^2013 | Cr | Beijing | N | Snowball | MSM | CESD-10≥10 | 105 | 48(45.7) |
| Jie Wu et al,^14^2011 | Cr | Guangzhou | 2010 | Web | MSM | CESD-20≥16 | 291 | 186(63.9) |
| Jinghua Li et al,^15^2016 | Cr | Chengdu | 2013 | NGO | HIVMSM | CESD-20≥16 | 321 | 179(55.8) |
| Ju Liu et al,^16^2012 | Cr | Foshan | 2010-2011 | RDS | MSM | SDS≥40 | 249 | 86(34.5) |
| Jun Tao et al,^17^2017 | Prospective cohort study | Beijing | 2013-2015 | VCT clinic | HIVMSM | HADS≥8 | 367 | 132(36.0) |
| Jun Tao et al,^18^2017 | RCT | Beijing | 2013-2015 | VCT clinic | HIVMSM | HADS≥8 | 228 | 98(43.0) |
| Ke Yun et al,^19^2018 | Cr | Shenyang | 2012 | VCT clinic | HNMSM | SDS≥42 | 140 | 31(22.4) |
| Lin Zhang et al,^20^2019 | Cr | Shanghai,Wuxi | 2018 | VCT clinic | HIVMSM | SDS≥42 | 267 | 247(92.5) |
| Liping Song et al,^21^2017 | Cr | Guilin | 2014 | Snowball | MSM | SDS≥42 | 400 | 113(28.3) |
| Liya Yu et al,^22^2017 | Cr | Liaoning | 2008-2009 | RDS | MSM | SDS≥42 | 807 | 267(33.1) |
| Peng Zhang et al,^23^2019 | Cr | Shanghai | 2017 | NGO | HIVMSM | MOS-HIV | 420 | 76(18.1) |
| Pengsheng Li et al,^24^2017 | Cr | Seven provinces^b^ | 2014-2015 | Cluster | YSMM | DSRSC≥15 | 2483 | 802(32.3) |
| Phoenix Kit-han Mo et al,^25^2018 | Cr | Chengdu | N | NGO | HIVMSM | DASS≥10 | 225 | 109(48.4) |
| Ping Wang et al,^26^2016 | Cr | Tianjin | 2015 | VCT clinic | YSMM | Depression subscale of SCL－90≥2 | 50 | 16(32.0) |
| Rong Pan et al,^27^2017 | Cr | Shanghai | 2014-2015 | Multiple methods | HIVMSM | CESD-12≥9 | 505 | 235(46.5) |
| Rui Li et al,^28^2016 | Cr | Shanghai | 2014 | Snowball | MSM | CESD-20≥16 | 547 | 285(52.1) |
| Shan Hu et al,^29^2016 | Cr | Chongqing,Sichuan | 2013-2014 | Multiple methods | MSM | CESD-20≥16 | 854 | 389(45.6) |
| Tingting Jiang et al,^30^2016 | Cr | Hangzhou,Wenzhou | 2014 | VCT clinic | MSM | SDS≥40 | 570 | 259(45.4) |
| Wei Sun et al,^31^2014 | Cr | Liaoning | 2010-2011 | VCT clinic | HIVMSM | CESD-20≥16 | 436 | 309(70.9) |
| Wei Xiong et al,^32^2018 | Cr | Chengdu | 2017-2018 | RDS | MSM | SDS≥42 | 103 | 37(35.9) |
| Wei Zhou et al,^33^2015 | Cr | Ningbo | 2014 | RDS | MSM | CESD-20≥16 | 126 | 24(19.1) |
| Weiming Sun et al,^34^2014 | Cr | Nanchang | 2013 | VCT clinic | HIVMSM | SDS≥42 | 65 | 41(63.1) |
| Xiaohong Pan et al,^35^2018 | Cr | Wenzhou | 2013-2014 | RDS | MSM | CESD-20≥16 | 454 | 243(53.5) |
| Xiaojuan Gao et al,^36^2018 | Cr | Kunming | 2016 | NGO | MSM | SDS≥42 | 400 | 138(34.5) |
| Xiaoerun Tao et al,^37^2009 | Cr | Shandong | 2007 | Venue based | MB | SDS≥40 | 116 | 79(68.1) |
| Xiaoxue Peng et al,^38^2017 | Cr | Shenzhen | N | RDS | MSM | Anxiety and depression emotion scale≥40 | 596 | 307(51.5) |
| Xiaoyou Su et al,^39^2018 | Cr | Jiangsu | 2013-2014 | Multiple methods | HN/UnMSM | CESD-10 >20 | 507 | 136(26.8) |
| Xuemei Pan et al,^40^2016 | Cr | Liangzhou | 2014-2015 | Multiple methods | MSM | CESD-20≥16 | 312 | 159(51.0) |
| Yan Xie et al,^41^2018 | Cr | Shanghai | 2015 | VCT clinic | MSM | CESD-12≥9 | 738 | 286(38.8) |
| Yeen Huang et al,^42^ 2018 | Cr | Mainland China | 2014-2015 | Cluster | YSMM | DSRSC≥15 | 15066 | 3657(23.9) |
| Yi Wang et al,^43^2010 | Cr | Mianyang | 2008 | RDS | MSM | CESD-20≥16 | 201 | 76(37.8) |
| Yi Wang et al,^44^2018 | Cr | Mianyang | 2015-2017 | Snowball | YSMM | SDS≥40 | 356 | 118(33.2) |
| Yile Wu et al,^45^2014 | Cr | Anhui | 2013 | VCT clinic | HIVMSM | CESD-20≥22 | 184 | 79(42.9) |
| Ying Hu et al,^46^2018 | Cr | Twelve cities^c^ | 2013-2014 | Multiple methods | HN/UnMSM | CESD-20≥16 | 1809 | 682(37.7) |
| Ying Liu et al,^47^2017 | Cr | Central region | 2013-2014 | VCT clinic | HIVMSM | PHQ-9≥10 | 321 | 132(41.1) |
| Yingjun Zheng et al, ^48^2004 | Cr | Central region | 2004 | Venue based | MSM | SDS≥40 | 167 | 96(57.5) |
| Yong Yu et al,^49^2016 | Cr | Guangzhou | 2014-2015 | RDS | MSM | DASS＞6.03 | 420 | 163(38.8) |
| Yuji Feng et al,^50^2010 | Cr | Chengdu | 2007 | Snowball | MSM | CESD-20≥16 | 513 | 275(53.7) |
| Yunhui Liu et al,^51^2015 | Cr | Beijing | 2013 | VCT clinic | HIVMSM | CESD-20≥16 | 76 | 33(43.4) |
| Zhen Li et al,^52^2016 | Cr | Beijing | 2014 | VCT clinic | HIVMSM | CESD-20≥16 | 266 | 146(54.9) |

Abbreviation: Cr, Cross-sectional study; RCT, randomize control trial; N, Not report; NGO, [Non-governmental](javascript:;) [organization](javascript:;); VCT, HIV Voluntary Counseling & Testing; RDS, Respondent-driven sampling; MSM, Men who have sex with Men; HNMSM, HIV-negative MSM; UnMSM, Unknown HIV status MSM; HIVMSM, HIV-positive MSM; YSMM, young sexual minority male; MB, Money boy; CBDI-Ⅱ, Chinese version of Beck Depression Inventory; SDS, Self-Rating Depression scale; CESD, Center for Epidemiological Studies Depression Scale; HADS, Hospital Anxiety and Depression Scale; MOS-HIV, Medical Outcomes Study HIV Health Survey; DSRSC, Depression Self-Rating Scale for Children; PHQ, Patient Health Questionnaire; DASS: Depression Anxiety and Stress scale;SCL-90: Symptom Checklist 90;

^a^ Studies are ordered alphabetically by authors name.

^b^ Seven provinces including Guangdong, Liaoning, Shandong, Hunan, Shanxi, Chongqing and Guizhou

^c^ Twelve cities including Chongqing (Chongqing City, Wanzhou District), Sichuan (Mianyang, Nanchong, Suining, Yibin and Luzhou cities), Xinjiang (Wulumuqi and Yili cities) and Guangxi (Nanning, Liuzhou and Beihai cities).

**Selected characteristics of the 27 studies reporting anxiety**

| Study | Study design | Location | Survey year | Sampling methods | Study population | Instrument and Cut-off Score | | Sample size | | Men,  No.(%) |
| --- | --- | --- | --- | --- | --- | --- | --- | --- | --- | --- |
| Dongliang Li et al, ^3^2015 | Cr | Shanghai | 2012 | Snowball | MSM | GAD-7≥10 | 307 | | 89(28.9) | |
| Fang Chen et al, ^5^2015 | Cr | Chengdu,Chongqing,Guangzhou | 2012-2013 | Snowball | HIVMSM | SAS≥40 | 541 | | 136(25.1) | |
| Fengrong Huang et al, ^6^2010 | Cr | Shanghai | N | Snowball | YSMM | SAS≥40 | 52 | | 14(26.9) | |
| Huan He et al,^11^2015 | Cr | Shanghai,Chengdu | 2013 | Snowball | HIVMSM | SAS≥40 | 308 | | 119(38.6) | |
| Jia Yu et al, ^13^2013 | Cr | Beijing | N | Snowball | MSM | SAS≥40 | 105 | | 36(34.3) | |
| Jinwei Lv et al,^53^2014 | Cr | Wuhu | 2012 | VCT clinic | MSM | SAS≥40 | 162 | | 75(46.3) | |
| Jun Tao et al, ^17^2017 | Prospective cohort | Beijing | 2013-2015 | N | HIVMSM | HADS≥8 | 367 | | 154(42.0) | |
| Jun Tao et al, ^18^2017 | RCT | Beijing | 2013-2015 | N | HIVMSM | HADS≥8 | 228 | | 111(48.7) | |
| Lin Zhang et al, ^20^2019 | Cr | Shanghai,Wuxi | 2018 | VCT clinic | HIVMSM | SAS≥40 | 267 | | 154(57.6) | |
| Liping Song et al, ^21^2017 | Cr | Guangxi | 2014 | Snowball | MSM | SAS≥40 | 400 | | 98(24.5) | |
| Liya Yu et al, ^22^2017 | Cr | Liaoning | 2008-2009 | RDS | MSM | SAS≥40 | 807 | | 105(13.0) | |
| Peng Zhang et al, ^23^2019 | Cr | Shanghai | 2015 | VCT clinic | YSMM | MOS-HIV | 420 | | 143(34.0) | |
| Ping Wang et al,^26^2016 | Cr | Tianjin | 2015 | VCT clinic | YSMM | Anxiety subscale of SCL－90≥2 | 50 | | 13(26.0) | |
| Rui Li et al,^28^2016 | Cr | Shanghai | 2014 | Snowball | MSM | GAD-7≥10 | 547 | | 67(12.2) | |
| Shan Hu et al, ^29^2016 | Cr | Chongqing,Sichuan | 2013-2014 | Multiple methods | MSM | SAS≥40 | 854 | | 273(32.0) | |
| Tingting Jiang et al, ^30^2016 | Cr | Hangzhou,Wenzhou | 2014 | VCT clinic | MSM | SAS≥40 | 570 | | 137(24.0) | |
| Wei Sun et al, ^31^2014 | Cr | Liaoning | 2010-2011 | VCT clinic | HIVMSM | SAS≥40 | 436 | | 194(44.5) | |
| Weiming Sun et al,^34^2014 | Cr | Nanchang | 2013 | VCT clinic | HIVMSM | SAS≥40 | 65 | | 21(32.3) | |
| Xiaorun Tao et al, ^37^2009 | Cr | Shandong | 2007 | Venue based | MB | SAS≥40 | 116 | | 54(46.6) | |
| Xiaoxue Peng et al, ^38^2017 | Cr | Shenzhen | N | RDS | MSM | Anxiety and depression emotion scale≥50 | 596 | | 217(36.4) | |
| Yi Wang et al, ^44^2018 | Cr | Mianyang | 2015-2017 | Snowball | YSMM | SAS≥40 | 356 | | 84(23.6) | |
| Yile Wu et al, ^45^2014 | Cr | Anhui | 2013 | VCT clinic | HIVMSM | SAS≥40 | 184 | | 59(32.1) | |
| Ying Hu et al,^46^2018 | Cr | Twelve cities^c^ | 2013-2014 | Multiple methods | HN/UnMSM | SAS≥40 | 1809 | | 463(25.6) | |
| Ying Liu et al,^47^2017 | Cr | Central region | 2013-2014 | VCT clinic | HIVMSM | GAD-7≥10 | 321 | | 101(31.5) | |
| Yingjun Zheng et al, ^48^2004 | Cr | Central region | 2004 | Venue based | MSM | SAS≥40 | 167 | | 76(45.5) | |
| Yong Yu et al,^49^2016 | Cr | Guangzhou | 2014-2015 | RDS | MSM | DASS≥5.26 | 420 | | 162(38.6) | |
| Yunhui Liu et al,^51^2015 | Cr | Beijing | 2013 | VCT clinic | HIVMSM | GAD-7≥5 | 76 | | 32(42.1) | |

Abbreviations: GAD-7, General Anxiety Disorder-7 item; SAS, Self-Rating Anxiety Scale.

^c^ Twelve cities including Chongqing (Chongqing City, Wanzhou District), Sichuan (Mianyang, Nanchong, Suining, Yibin and Luzhou cities), Xinjiang (Wulumuqi and Yili cities) and Guangxi (Nanning, Liuzhou and Beihai cities).

**Selected characteristics of the 23 studies reporting suicidal ideation**

| Study | Study design | Location | Survey year | Sampling methods | Study population | Recall time | Sample size | Men,  No.(%) |
| --- | --- | --- | --- | --- | --- | --- | --- | --- |
| Dandan Zhang et al,^2^2018 | Cr | Ningbo | N | VCT clinic | MSM | 7 days | 516 | 93(18.0) |
| Fang Chen et al, ^5^2015 | Cr | Chengdu,Chongqing,Guangzhou | 2012-2013 | Snowball | HIVMSM | 12 months | 541 | 116(21.4) |
| Hongquan Chen et al,^54^2011 | Cr | Nine cities | 2006 | Snowball | MSM | Lifetime | 1470 | 297(20.2) |
| Hongquan Chen et al,^55^2015 | Cr | Nine cities | 2001-2005 | Snowball | MSM | Lifetime | 1530 | 398(26.0) |
| Huijuan Mu et al,^56^2016 | Cr | Liaoning | 2008-2009 | RDS | MSM | 12 months | 807 | 148(18.3) |
| Jia Yu et al, ^13^2013 | Cr | Beijing | N | Snowball | MSM | 7 days | 105 | 10(9.5) |
| Jing Li et al,^57^2018 | Cr | Foshan | 2010-2011 | RDS | MSM | Lifetime | 249 | 84(33.7) |
| Lijian Xue et al,^58^2011 | Cr | Jiangsu | 2010 | Web | MSM | Lifetime | 838 | 160(19.1) |
| Liya Yu et al, ,^22^2017 | Cr | Liaoning | 2008-2009 | RDS | MSM | 12 months | 807 | 78(9.7) |
| Phoenix Kit-han Mo et al,^25^2018 | Cr | Chengdu | N | NGO | HIVMSM | Since HIV diagnosis | 225 | 108(48.0) |
| Rui Li et al, ^28^2016 | Cr | Shanghai | 2014 | Snowball | MSM | 12 months | 547 | 58(10.6) |
| Wu Zheng et al,^59^2012 | Cr | Wuhan | N | Snowball | MSM | Lifetime | 253 | 47(18.6) |
| Xiaohong Pan et al,^35^2018 | Cr | Wenzhou | 2013-2014 | RDS | MSM | 12 months | 454 | 73(16.1) |
| Yang Li et al,^60^2010 | Cr | Nine cities | 2005-2006 | Snowball | MSM | N | 1811 | 383(21.1) |
| Yeen Huang et al,^61^ 2017 | Cr | Mainland China | 2014-2015 | Cluster | YSMM | 12 months | 2483 | 769(31.0) |
| Yi Wang et al,^62^2011 | Cr | Mianyang | 2007 | RDS | MSM | 6 months | 201 | 18(9.0) |
| Yi Wang et al,^44^2018 | Cr | Mianyang | 2015-2017 | Snowball | YSMM | 7 days | 347 | 70(20.2) |
| Yile Wu et al, ^45^2014 | Cr | Anhui | 2013 | VCT clinic | HIVMSM | 6 months | 184 | 57(31.0) |
| Yilu Qin et al,^63^2017 | Cr | Mainland China | 2015 | Web | MSM | N | 341 | 119(35.0) |
| Ying Liu et al, ^47^2017 | Cr | Central region | 2013-2014 | VCT clinic | HIVMSM | Lifetime | 321 | 96(29.9) |
| Yingjun Zheng et al, ^48^2004 | Cr | Central region | 2004 | Venue based | MSM | 7 days | 174 | 69(39.7) |
| Yong Yu et al,^64^2017 | Cr | Guangzhou | 2014 | RDS | MSM | 12 months | 420 | 123(29.3) |
| Yuanyuan Wang et al,^65^2018 | Cr | Shenzhen | 2015-2017 | VCT clinic | HIVMSM | 1 month | 410 | 42(10.3) |

**Selected characteristics of the 9 studies reporting suicide plan**

| Study | Study design | Study Location | Survey year | Sampling methods | Study population | Recall time | Sample size | Men,  No.(%) |
| --- | --- | --- | --- | --- | --- | --- | --- | --- |
| Hongbo Zhang et al,^66^2007 | Cr | Hefei | 2005 | RDS | MSM | 1 month | 228 | 9(3.9) |
| Hongquan Chen et al, ^55^2015 | Cr | Nine cities | 2001-2005 | Snowball | MSM | Lifetime | 1530 | 398(26.0) |
| Huijuan Mu et al, ^56^2016 | Cr | Liaoning | 2008-2009 | RDS | MSM | 12 months | 807 | 148(18.3) |
| Liya Yu et al, ^22^2017 | Cr | Liaoning | 2008-2009 | RDS | MSM | 12 months | 807 | 78(9.7) |
| Rui Li et al, ^28^2016 | Cr | Shanghai | 2014 | Snowball | MSM | 12 months | 547 | 58(10.6) |
| Yi Wang et al, ^62^2011 | Cr | Mianyang | 2007 | RDS | MSM | 6 months | 201 | 18(9.0) |
| Ying Liu et al, ^47^2017 | Cr | Central region | 2013-2014 | VCT clinic | HIVMSM | Lifetime | 321 | 96(29.9) |
| Yong Yu et al, ^64^2017 | Cr | Guangzhou | 2014 | RDS | MSM | 12 months | 420 | 123(29.3) |
| Yuanyuan Wang et al, ^66^2018 | Cr | Shenzhen | 2015-2017 | VCT clinic | HIVMSM | 1 month | 410 | 42(10.3) |

**Selected characteristics of the 19 studies reporting suicide attempt**

| Study | Study design | Study Location | Survey year | Sampling methods | Study population | Recall time | Sample size | Men,  No.(%) |
| --- | --- | --- | --- | --- | --- | --- | --- | --- |
| Guanzhi Chen et al,^67^2012 | Cr | Nine cities | 2001-2005 | Snowball | MSM | N | 714 | 128(17.9) |
| Hongbo Zhang et al, ^66^2007 | Cr | Hefei | 2005 | RDS | MSM | 1 month | 228 | 9(3.9) |
| Huijuan Mu et al, ^56^2016 | Cr | Liaoning | 2008-2009 | RDS | MSM | 12 months | 807 | 148(18.3) |
| Jia Yu et al, ^13^2013 | Cr | Beijing | N | Snowball | MSM | 7 days | 105 | 10(9.5) |
| Jing Li et al, ^57^2018 | Cr | Foshan | 2010-2011 | RDS | MSM | Lifetime | 249 | 84(33.7) |
| Lijian Xue et al,^58^2011 | Cr | Jiangsu | 2010 | Web | MSM | Lifetime | 838 | 160(19.1) |
| Yi Wang et al, ^44^2018 | Cr | Mianyang | 2015-2017 | Snowball | YSMM | N | 356 | 32(9.1) |
| Liya Yu et al, ^22^2017 | Cr | Liaoning | 2008-2009 | RDS | MSM | 12 months | 807 | 78(9.7) |
| Phoenix Kit-han Mo et al,^25^2018 | Cr | Chengdu | N | NGO | HIVMSM | Since HIV diagnosis | 225 | 108(48.0) |
| Rui Li et al, ^28^2016 | Cr | Shanghai | 2014 | Snowball | MSM | 12 months | 547 | 58(10.6) |
| Wu Zheng et al,^59^2012 | Cr | Wuhan | N | Snowball | MSM | Lifetime | 253 | 47(18.6) |
| Yang Li et al, ^60^2010 | Cr | Nine cities | 2005-2006 | Snowball | MSM | N | 1811 | 383(21.1) |
| Yeen Huang et al, ^61^2017 | Cr | Mainland China | 2014-2015 | Cluster | YSMM | 12 months | 2483 | 254(10.2) |
| Yeen Huang et al,^42^2018 | Cr | Mainland China | 2014-2015 | Cluster | YSMM | 12 months | 15066 | 653(4.2) |
| Yi Wang et al, ^62^2011 | Cr | Mianyang | 2007 | RDS | MSM | 6 months | 201 | 18(9.0) |
| Ying Liu et al, ^47^2017 | Cr | Central region | 2013-2014 | VCT clinic | HIVMSM | Lifetime | 321 | 96(29.9) |
| Yile Wu et al, ^45^2014 | Cr | Anhui | 2013 | VCT clinic | HIVMSM | 6 months | 184 | 57(31.0) |
| Yingjun Zheng et al, ^48^2004 | Cr | N | 2004 | Venue based | MSM | 7 days | 174 | 69(39.7) |
| Yong Yu et al, ^53^2017 | Cr | Guangzhou | 2014 | RDS | MSM | 12 months | 420 | 123(29.3) |

**Reference**

1. Shiu C-S, Chen Y-C, Tseng P-C, et al. Curvilinear relationship between depression and unprotected sexual behaviors among men who have sex with men. *J Sex Med.* 2014;11(10):2466-2473.

2. Zhang D-D, Hong H, Jiang H-B. Depression in men who have sex with men in Ningbo. *Prev Med.* 2018;30(5):442-445.

3. Li D, Li C, Wang Z, Lau JTF. Prevalence and associated factors of unprotected anal intercourse with regular male sex partners among HIV negative men who have sex with men in China: a cross-sectional survey. *PloS One.* 2015;10(3):e0119977-e0119977.

4. Wong FY, Huang ZJ, He N, et al. HIV risks among gay- and non-gay-identified migrant money boys in Shanghai, China. *AIDS Care.* 2008;20(2):170-180.

5. Chen F, Lin X-J, Wang X-D, et al. Prevalence rates of depression and anxiety in HIV-infected men who have sex with men. *Chin Ment Health J.* 2015;29(4):251-257.

6. Huang FR, Huang H, Zhuang MH, et al. Investigations on social psychology and sexual behavior of 52 male homosexual adolescents in Shanghai. *Shanghai Jiao Tong Da Xue Xue Bao Yi Xue Ban.* 2010;30(5):581-584.

7. Guozheng S. Study on depression status and related factors among men who have sex with men in Jiading District. *Chinese Primary Health Care.* 2014;28(4):65-67.

8. Yan H, Li X, Li J, et al. Association between perceived HIV stigma, social support, resilience, self-esteem, and depressive symptoms among HIV-positive men who have sex with men (MSM) in Nanjing, China. *AIDS Care.* 2019;31(9):1069-1076.

9. Wang H-Y, Wang N, Chu Z-X, et al. Intimate partner violence correlates with a higher HIV incidence among MSM: a 12-month prospective cohort study in Shenyang, China. *Sci Rep.* 2018;8(1):2879.

10. Yan H, Wong FY, Zheng T, et al. Social support and depressive symptoms among ‘money’ boys and general men who have sex with men in Shanghai, China. *Sex Health.* 2014;11(3):285-287.

11. He H, Zhang H, Ding F, et al. Risky sexual transmission behavior and its influencing factors among HIV-positive MSM population in Shanghai and Chengdu in China. *Chin J Epidemiol.* 2015;36(3):254-258.

12. Ibragimov U, Harnisch JA, Nehl EJ, et al. Estimating self-reported sex practices, drug use, depression, and intimate partner violence among MSM in China: A comparison of three recruitment methods. *AIDS Care.* 2017;29(1):125-131.

13. Yu J, Wang Z, Li C, Liu, D. Mental health status of MSM in Beijing. *Chin J AIDS STD.* 2013;19(4):241-243.

14. Wu J, Lu C, Hao Y, et al. Psychosocial status of MSM population in Guangzhou City. *Chin J Public Health.* 2011;27(4):476-478.

15. Li J, Mo PKH, Wu AMS, Lau JTF. Roles of self-stigma, social support, and positive and negative affects as determinants of depressive symptoms among HIV infected men who have sex with men in China. *AIDS Behav.* 2017;21(1):261-273.

16. Liu J, Gao Y-H, Liang Z-M, Li Y, Yang Y. Depressive symptoms and associated sexual behaviors among men who have sex with men in Foshan, Guangdong province. *Zhonghua Liu Xing Bing Xue Za Zhi.* 2012;33(5):483-487.

17. Tao J, Vermund SH, Lu H, et al. Impact of depression and anxiety on initiation of antiretroviral therapy among men who have sex with men with newly diagnosed HIV infections in China. *AIDS Patient Care STDS.* 2017;31(2):96-104.

18. Tao J, Qian H-Z, Kipp AM, et al. Effects of depression and anxiety on antiretroviral therapy adherence among newly diagnosed HIV-infected Chinese MSM. *AIDS.* 2017;31(3):401-406.

19. Yun K, Chu Z, Hu Q, et al. Association between psychological disorders, depression and the CD4 level of HIV negative MSM in Shenyang. *Chin J AIDS STD.* 2018;24(11):1118-1123.

20. Zhang L, Zhou Y, et al. Anxiety states of homosexual AIDS men who have heterosexual marriage. *Shanghai Journal of Preventive Medicine.* 2019;31(4):294-300.

21. Song L, Zhang Y, Lan G, et al. The impacting factor of peer education among MSM. *Chin J Dis Control Prev.* 2017;21(11):1132-1135.

22. Yu L, Li Y, Liu L, et al. Association of recent gay-related stressful events and emotional distress with suicidal behaviors over 12 months in Chinese men who have sex with men. *Asia Pac Psychiatry.* 2018;10(1):1758-5872.

23. Zhang P, Gao J, Wang Y, Sun Q, Sun X. Effect of chronic disease self-management program on the quality of life of HIV-infected men who have sex with men: an empirical study in Shanghai, China. *Int J Health Plann Manage.* 2019;34:1055-1064.

24. Li P, Huang Y, Guo L, et al. Is sexual minority status associated with poor sleep quality among adolescents? Analysis of a national cross-sectional survey in Chinese adolescents. *BMJ Open.* 2017;7(12):e017067.

25. Mo PK-H, Lau JT-F, Wu X. Relationship between illness representations and mental health among HIV-positive men who have sex with men. *AIDS Care.* 2018;30(10):1246-1251.

26. Wang P, Liu Z, Guo Y, Zhang X. Association between mental health and family environment among young male students who have sex with men. *Chin J Sch Health.* 2016;37(4):523-526.

27. Pan R, Kun C, Zheng H, et al. Depressive symptoms and related factors among HIV-positive men who have sex with men in Shanghai. *Fudan Univ J Medical Sci.* 2017;44:430-434.

28. Li R, Cai Y, Wang Y, Gan F, Shi R. Psychological pathway to suicidal ideation among men who have sex with men in Shanghai, China: a structural equation model. *J Psychiatr Res.* 2016;83:203-210.

29. Hu S, Zhong X-N, Wen X-Y, et al. Characteristics of anxiety and depression symptoms among gays and bisexual men with HIV negative. *Chinese Mental Health Journal.* 2016;30(3):213-219.

30. Jiang T, Wang H, Zhou X, Ma Q. Depressive, anxiety symptoms and related influential factors among men who have sex with men in Zhejiang. *Chin J AIDS STD.* 2016;22:357-360.

31. Sun W, Wu M, Qu P, Lu C, Wang L. Psychological well-being of people living with HIV/AIDS under the new epidemic characteristics in China and the risk factors: a population-based study. *Int J Infect Dis.* 2014;28:e147-e152.

32. Xiong W, Zhang R, Liu Y, Yin L. Depression of gay college students in Chengdu and its influencing factors. *Chin J Sch Health.* 2018;39(8):1239-1241.

33. Wang Y, Xu J, Zhang G, Yang H, Fan J. Depressive symptoms and related factors in MSM. *Chinese Mental Health Journal.* 2010;24(5):366-367,369.

34. Sun W, Yuanye F, Xu D, Cheng X, Zou Q, Lou Q. The anxiety and depression of male homosexual with HIV/AIDS in Nanchang City: current situation and related factors. *Chin J Dis Control Prev.* 2014;18:1143-1146.

35. Pan X, Li R, Ma Q, et al. Sexual risk behaviour, sexual victimisation, substance use and other factors related to depression in men who have sex with men in Wenzhou, China: a cross-sectional study. *BMJ Open.* 2018;8(4)-2016-013512.

36. Gao X, Li Y, et al. Depression and related influential factors among men who have sex with men in Kunming. *Chin J AIDS STD.* 2018;24:170-173.

37. Tao X, Zheng W, Liu J, et al. Mental status of money boys in Shandong province. *Chin J Public Health.* 2009;25(9):1092-1093.

38. Peng X, Qi J, Chen M. State of anxiety and depression and related influencing factors among men who have sex with men in Shenzhen. *Chin J AIDS STD.* 2017;7:630-633.39.

39. Su X, Zhou AN, Li J, et al. Depression, loneliness, and sexual risk-taking among HIV-negative/unknown men who have sex with men in China. *Arch Sex Behav.* 2018;47(7):1959-1968.

40. Pan X, Zheng W, Huang J, Deng Y, Chen Z. Investigation of current status of depression, quality of life and influencing factors among men who have sex with men in Lianzhou. *Jiangsu J Prev Med.* 2016;27(1):44-47.

41. Xie Y, Yang Y. Analysis on depressive symptoms and related factors of men who have sex with men attending HIV voluntary counseling and testing clinics in Shanghai. *Chinese Journal of Disease Control and Prevention.* 2018;22(12):1248-1251.

42. Huang Y, Li P, Lai Z, et al. Chinese sexual minority male adolescents’ suicidality and body mass index. *Int J Environ Res Public Health.* 2018;15(11):2558.

43. Wang Y, Xu J, Zhang G, Yang H, Fan J. Relations between self-discrimination of MSM and sexual behavior and psychological factors. *Chin J Prev Med.* 2010;44(7):636-644.

44. Wang Y, Zhou W, Fan J, et al. Relationship of anxiety and depressive symptoms with social behavior among young men who have sex with men. *South China J Prev Med.* 2018;44(5):401-405.

45. Wu Y-L, Yang H-Y, Wang J, et al. Prevalence of suicidal ideation and associated factors among HIV-positive MSM in Anhui, China. *Int J STD AIDS.* 2015;26(7):496-503.

46. Hu Y, Zhong X-N, Peng B, et al. Comparison of depression and anxiety between HIV-negative men who have sex with men and women (MSMW) and men who have sex with men only (MSMO): a cross-sectional study in Western China. *BMJ Open.* 2019;9(1):e023498-e023498.

47. Liu Y, Niu L, et al. Emotional problems among newly diagnosed HIV-positive men with homosexual sex behaviors. *Chinese Mental Health Journal*. 2017;31(6):471-477.

48. Zheng Y, Xu J, Zhao B, Zhang H. Psychosocial distinction of men who have sex with men. *Chin Behav Med Sci.* 2004;13(6):655-657.

49. Yu Y, Li Y, Wang P. Gay men’s gender roles and mental health in Guangzhou. *Chin J Health Psychol.* 2016;24(11):1618-1621,1622.

50. Feng Y, Wu Z, Detels R, et al. HIV/STD prevalence among men who have sex with men in Chengdu, China and associated risk factors for HIV infection. *J Acquir Immune Defic Syndr.* 2010;53 Suppl 1:S74-S80.

51. Liu Y, Liu X. Investigation and analysis of mental depression and anxiety of HIV/AIDS patients in MSM. *Chin J Prev Med.* 2015;16(7):578-580.

52. Li Z, Hsieh E, Morano JP, Sheng Y. Exploring HIV-related stigma among HIV-infected men who have sex with men in Beijing, China: a correlation study. *AIDS Care.* 2016;28(11):1394-1401.

53. Lv J, Cheng G, Cheng Z. Investigation on anxiety and intervention effect among men who have sex with men in Wuhu City. *Occup and Health.* 2014;30(13):1836-1838.

Zheng Y, Xu J, Zhao B, Zhang H. Psychosocial distinction of men who have sex with men. *Chin Behav Med Sci.* 2004;13(6):655-657.

54. Chen H, Zhang B, Li X. Study on high-risk behaviour and suicide associated risk factors related to HIV/AIDS among gay or bisexual men. *Chin J Epidemiol.* 2011;32(10):983-986.

55. Chen H, Li Y, Wang L, Zhang B. Causes of suicidal behaviors in men who have sex with men in China: a national questionnaire survey. *BMC Public Health.* 2015;15:91.

56. Mu H, Li Y, Liu L, et al. Prevalence and risk factors for lifetime suicide ideation, plan and attempt in Chinese men who have sex with men. *BMC Psychiatry.* 2016;16:117.

57. Li J, Li Y, Liang Z, et al. Association between impulsivity, aggression, and suicide among men who have sex with men (MSM) in Foshan City. *Chinese Journal of AIDS and STD.* 2018;24(11):1110-1114.

58. Xue L, Xu Y, Hong Z, Shi J, Zhao X, Zhu W. An online survey of HIV related knowledge and high risk behaviors, social pressure and their influencing factors among MSM. *Chin J AIDS STD.* 2011;17(3):324-327.

59. Zheng W, Wu C, Zhang B, Pi Q, Liu W. Analysis on influencing factors of suicide behavior among “MSM” in Wuhan. *J Public Health Prev Med.* 2012;23(1):51-55.

60. Li Y, Li X, Zang Y, Wang L, Chen G, Yu Z. Behavioral characteristics of men who have sex with men with sadomasochism associated with bleeding. *Chin J Epidemiol.* 2010;31(2):142-145.

61. Huang Y, Li P, Guo L, et al. Sexual minority status and suicidal behaviour among Chinese adolescents: a nationally representative cross-sectional study. *BMJ Open.* 2018;8(8):e020969.

62. Wang Y, Xu J, Zhang G, Yang H, Fan J. Logistic analysis for relatively risky factors of suicidal behaviors of MSM. *Journal of Modern Preventive Medicine.* 2011;38(12):2220-2223.

63. Qin Y, Tang W, Nowacki A, et al. Benefits and potential harms of human immunodeficiency virus self-testing among men who have sex with men in China: an implementation perspective. *Sex Transm Dis.* 2017;44(4):233-238.

64. Yu Y. Health and life satisfaction for Chinese gay men in Guangzhou, China. *J Cent South Univ.* 2017;42(12):1407-1416.

65. Wang Y-Y, Dong M, Zhang Q, et al. Suicidality and clinical correlates in Chinese men who have sex with men (MSM) with HIV infection. *Psychol Health Med.* 2019;24(2):137-143.

66. Zhang H, Zheng Y, Xu J, et al. Relationship between suicide behaviors and social character among men having sex with men in Hefei. *Chin J Public Health.* 2007;23(9):1027-1029.

67. Chen G, Li Y, Zhang B, et al. Psychological characteristics in high-risk MSM in China. *BMC Public Health.* 2012;12:58.
